# Evolutionary patterns of the mitochondrial control region in vertebrates: A large-scale comparative analysis

**DOI:** 10.1371/journal.pone.0353555

**Published:** 2026-07-10

**Authors:** Mauricio Ochoa Capera, Natalia S. Medina, Paula Montaña-Lozano, Manuela Moreno-Carmona, Antonio Baeza, Carlos Prada-Quiroga

**Affiliations:** 1 Departamento de Biología, Facultad de Ciencias, Universidad del Tolima, Tolima, Colombia; 2 Department of Biological Sciences, Clemson University, Clemson, South Carolina, United States of America; Griffith University, AUSTRALIA

## Abstract

The mitochondrial control region (CR) is the largest non-coding region in the vertebrate mitogenome and contains essential elements for replication and transcription. Despite its functional relevance, its evolutionary dynamics remain poorly understood. Here, we analyzed 5,235 complete vertebrate CRs spanning 11 classes to investigate how conserved sequences blocks (CSBs) and Extended Termination-Associated sequences (ETAS) shaped CR evolution. We hypothesized that CR length is positively associated with repeat accumulation, with tetrapods exhibiting longer and more complex CRs than fishes, while core elements remain conserved. Our analyses revealed marked inter- and intra-class variability, with longer CRs in tetrapods (1,283.27 ± 489.6 bp) than in fishes (969.25 ± 239.5 bp). Duplication events were restricted to tetrapods, especially birds and reptiles. Nucleotide composition was heterogeneous among orders, and structural divergence of CSBs was inferred across lineages. Repetitive elements were present in ~43% of CRs, with their abundance strongly correlated with CR length. Importantly, longer CRs were associated with higher GC content and greater variation in copy number of ETAS and CSBs. These results demonstrate that mitogenome CR expansion in vertebrates is largely driven by repeat proliferation, whereas key motifs required for replication and transcription are retained. We further identify lineage-specific trends, including pronounced CR elongation in amphibians and reptiles, contrasted with progressive reduction and simplification in birds and mammals. Our study provides the first comprehensive comparative framework of vertebrate CR evolution, highlighting how repetitive elements, conserved motifs, and nucleotide composition jointly contribute to both functional regulation and lineage-specific diversification.

## 1. Introduction

The vertebrate mitochondrial genome (mitogenome) is a circular molecule, typically 14–20 kbp in length that encodes for 37 genes (13 proteins: *atp6*, *atp8*, *cob*, *cox1–3*, *nad1–6*, *nad4L*; 22 transfer RNAs, 2 ribosomal RNA: *rrnS*, *rrnL*) and contains two non-coding regions; the L-strand origin replication (OL) and the Control Region (CR, or D-loop region) [1]. The length and gene order in vertebrate mitochondrial genomes was previously thought to be relatively conserved compared to those of invertebrates [1–4]. However, a recent study has shown relatively high architectural diversity of mitogenomes within vertebrate classes [5]. The later study, however, focused only on gene rearrangements of coding sequences (CDS), tRNAs and rRNAs and not the CR.

The CR has several important roles in the biogenesis and function of mitochondria, including an origin of replication of the heavy-strand and promoters for transcription of all genes in each strand [4,6]. The CR is a triple-stranded region found in the major non-coding region (NCR) of many vertebrate mitogenomes, located between the mt-tRNA genes coding for Phenylalanine and Proline [6,7]. This mitochondrial region is variable in length and ranges from 880 to 1,400 bp in mammalian species [4], between 900–1,400 bp in reptiles [8,9], between 1,028 ± 900–1,581 bp in Aves [10], and with reports in frogs of up to 3,200 bp [11]. The length of the CR has been suggested to be driven by expansion of tandemly repeated sequences [12,13]; however, comparative studies to test if indeed CR variability is controlled by the expansion of repetitive sequences in a manner analog to that reported for nuclear genomes in eumetazoans [8,14,15] are lacking.

Due to advances in sequencing technology, hundreds of vertebrate mitogenomes are currently available in open access databases; yet, for about half of these public-available mitogenomes, the CR has only been partially assembled or it is lacking. This is mostly due to problems during the bioinformatics assembly of this region, in turn, driven by the presence and high content of repetitive elements, most often microsatellites and short tandem repeats [5]. Although the length of the CR is variable in vertebrates mitogenomes, it does exhibits structural organization; short blocks of conserved sequences that are interspersed among more hypervariable regions (with higher mutation rate) have been detected in different vertebrates [4,8]. These conserved structural elements have been divided into three domains: i) extended termination-associated sequences (TAS/ETAS), adjacent to the Pro-tRNA gene and associated with termination of the newly H-strand during replication; ii) central conserved domain (CD) region usually located between TAS and CSB, which contains several areas of highly conserved sequences; and iii) conserved sequence blocks (CSBs) serve as transcription promoters [4,8].

Previous studies on the evolution of CSB in vertebrate mitogenomes have focused on specific taxonomic groups. For example, in turtles, comparative analysis has shown that the CD region is more conserved than the TAS and CSB domains [8,16]. However, TAS and CSB still exhibit variation both in terms of position within the CR and identity and number of conserved elements. For example, TAS vary between 1–2 copies among mammals and turtles [4,8,16]; while CSB in mammals can vary between 2 and 5 copies [4], 3 copies in birds [10] and in turtles, only 3–4 copies have been reported [8]. No systematic vertebrate-wide analysis has yet assessed the conservation and variability of CR domains and sequence blocks, key elements underlying mitochondrial replication and transcription.

Although approximately 7,000 vertebrate mitogenomes are currently available in GenBank (https://www.ncbi.nlm.nih.gov/), detailed studies of the mitochondrial control region (CR) have generally focused on a limited number of taxa and relatively small datasets. Consequently, the evolutionary diversity of this key regulatory region remains incompletely understood, and the extent of variation in its conserved and hypervariable structural elements across vertebrate lineages has yet to be comprehensively characterized.

In this study, we have compared the organization of the mitochondrial genome in vertebrates to shed light on its evolution. We hypothesize that (I) variation in vertebrate CR length are related to events of insertion/deletion of repetitive elements, (II) there are taxa with greater variation in copy number and composition of ETAS and CSBs than others; (III) despite CR variability, there are elements that are conserved throughout vertebrate evolution as we expect specific features of the CR to play most important roles on its translation and replication. We have compared a total of 5,235 complete CR sequences obtained from the NCBI’s GenBank database. We have identified possible conserved sites for TAS, CD and CSB domains and characterized the composition of the hypervariable regions in each CR to systematically characterize its structural variability within and among the 11 vertebrate taxonomic classes. Additionally, CR features which are shared or not among vertebrate taxonomic classes have been identified and their implications in both functional and evolutionary aspects are discussed in detail.

## 2. Materials and methods

### 2.1. CR sequences of vertebrate species

#### 2.1.1. Dataset retrieval.

We retrieved complete vertebrate mitochondrial genomes from the NCBI Organelle Genome Resource database (https://www.ncbi.nlm.nih.gov/genome/browse#!/organelles/) as of December 20, 2022. Searches were restricted to records annotated as “complete mitochondrial genome” in GenBank. A total of 5,363 mitogenomes representing 11 vertebrate classes; Mammalia (1093), Aves (770), Reptilia (314), Amphibia (241) and fish (Dipneusti (7), Actinopteri (2735), Cladistii (11), Holocephali (9), Elasmobranchii (170), Petromyzonti (11) and Myxini (2)), were initially downloaded. We note that although Aves and fish are not monophyletic groups [17,18], we have retained these taxa for our systematic review to facilitate comparison with older literature.

#### 2.1.2. Inclusion and exclusion criteria.

To ensure consistency across datasets, we excluded mitogenomes representing multiple strains of the same species (e.g., *Mus musculus* laboratory strains). After filtering, each species was represented by a single mitogenome. Sequences were further filtered based on the following criteria: i) availability of full mitochondrial genome sequence (no partial genomes included), ii) absence of ambiguous assembly annotations affecting the control region, and iii) exclusion of entries lacking sufficient annotation to identify control region boundaries. After applying all filters, 5,235 mitogenomes were retained for downstream analyses (Supplementary S1 Table). Heteroplasmy was not assessed because analyses were based on assembled mitochondrial genomes from GenBank, which represent consensus sequences and do not preserve underlying heteroplasmic variation. Mitochondrial molecular features, including CR length (bp), CR nucleotide composition, CR duplication events, number and types of repetitive elements in the CR, and presence of conserved structural elements were compared among classes and lower taxonomic groups (Order, Family, Genus and Species) using the classification of Mammal Species of the World https://www.gbif.org/es/dataset/672aca30-f1b5-43d3-8a2b-c1606125fa1b), Amphibian Species of the World (https://amphibiansoftheworld.amnh.org/), Birds of the World (https://birdsoftheworld.org/bow/home), Eschmeyer’s Catalog of Fishes (https://www.calacademy.org/scientists/projects/eschmeyers-catalog-of-fishes), and Integrated Taxonomic Information System (https://www.itis.gov/).

#### 2.1.3. Identification of putative CRs in unannotated genomes and quality filtering of CR sequences.

For mitogenomes lacking explicit CR annotation, we identified putative CRs using comparative genomics approaches. Whole mitochondrial genomes of closely related taxa were aligned using Clustal Omega [19] and NCBI–BLAST2Seq (https://blast.ncbi.nlm.nih.gov/Blast.cgi). Regions showing ≥70% sequence identity with orthologous CRs were considered putative CRs. To refine boundaries and confirm homology, multiple sequence alignments of extracted CR regions were performed. All sequence extraction and annotation curation were conducted using Geneious [20]. Putative CR sequences shorter than 300 bp were excluded from downstream analyses, based on previously reported minimum vertebrate CR lengths [4]. Additionally, taxonomic groups represented by fewer than two CR sequences were excluded from comparative analyses.

#### 2.1.4. Detection of duplication events.

CR duplication events were inferred based on the presence of multiple homologous CR-like regions within a single mitochondrial genome, identified through alignment-based comparison using Clustal Omega [19], NCBI–BLAST2Seq and annotation inspection.

### 2.2. Molecular characterization of the CR

#### 2.2.1. Global CR characterization.

Each mitochondrial control region (CR) was characterized by i) nucleotide composition, ii) strand asymmetry (AT and GC skew), iii) repetitive element content, and, iv) presence of conserved regulatory domains: **Domain I** corresponding to termination-associated sequences (TASs), **Domain II** corresponding to central domains (CSB − A to CSB − F) and, **Domain III** corresponding to CSB or right domains (CSB − 1 to CSB − 3). GC content and CR length were computed using Geneious [20]. Strand asymmetry was calculated as: AT skew = [A% − T%]/ [A% + T%] and GC skew = [G% − C%]/ [G% + C%] [2]. Correlations between AT and GC skew were evaluated using Pearson correlation in RStudio [21] with the package ‘FactoMineR’.

#### 2.2.2. Detection of tandem repeats.

Tandem repeats were identified using Tandem Repeats Finder (TRF) with default parameters unless otherwise specified (https://tandem.bu.edu/trf/trf.html) (S1 Table). Extracted features included repeat number, motif size and genomic position. Correlations between CR length and repeat number were assessed using Pearson correlation in RStudio [21] with the package ‘FactoMineR’ and package ‘ggplot2’ to visualize results, considering only genomes with a single repeat type to avoid potential confounding effects. Differences in CR length, GC content, and repeat number among taxonomic groups were tested using Kruskal-Wallis tests followed by Dunn’s post hoc comparisons with Bonferroni correction in RStudio [21].

#### 2.2.3. Identification of Extended Termination-Associated sequences (TAS/ETAS) region.

The TAS/ETAS region was identified using motif-based scanning of the canonical sequence ATG-9nt-CAT (coreTAS) as described by Jemt et al. [22]. Two IUPAC-based motif definitions were used: i) coreTAS_M: ATGNHHDHHSHVCAT (strict motif) and ii) coreTAS_LS: ATGNNNNNNNNNCAT (relaxed motif). Motif searches were performed using Fuzznuc (EMBOSS) [23], with default mismatch settings. A hierarchical search strategy was applied: coreTAS_M was searched first, followed by coreTAS_LS when no match was detected. Coordinates of detected motifs identified by Fuzznuc were extracted and parsed using custom scripts written in Python v3.10. The scripts used the Biopython package to process EMBOSS output files and retrieve motif positions, sequence orientation, and occurrence counts for downstream analyses. When multiple hits were present, all occurrences were retained for downstream analysis.

#### 2.2.4. Annotation of conserved sequences blocks (CSBs).

CSB motifs (CSB-A to CSB-F and CSB1-CSB3) were annotated using a combined approach: i) reference-guided motif identification based on previously described vertebrate CR studies (see S2 Table), and ii) homology-guided motif transfer for taxa lacking direct annotations, using the closest phylogenetically related order as reference. Motif scanning was performed using Fuzznuc (EMBOSS) [23]. A putative CSB was considered present when it matched ≥60% sequence identity to the reference motif. This threshold was chosen to accommodate known variability in regulatory mitochondrial motifs while retaining positional conservation.

#### 2.2.5. Alignment, comparative and phylogenetic analysis of CSB motifs.

CR sequences were aligned by taxonomic order using Clustal Omega [19]. Alignments were used to refine CSB boundaries and assess positional conservation across taxa. Conserved motif patterns were further evaluated at the class level using multiple sequence alignment inspection. To explore evolutionary relationships among CSB variants, maximum-likelihood analyses were conducted in MEGA [24]. The best-fit nucleotide substitution model was selected using the Model Selection function implemented in MEGA according to the Bayesian Information Criterion (BIC). Node support was assessed using 1,000 bootstrap replicates. The resulting trees were used to explore broad patterns of motif similarity among vertebrate lineages rather than to infer species-level phylogenetic relationships. All identified CSB motifs and their coordinates are provided in Supplementary S4 Table.

## 3. Results

### 3.1. Dataset composition and global features of vertebrate CRs

**Fig 1:**
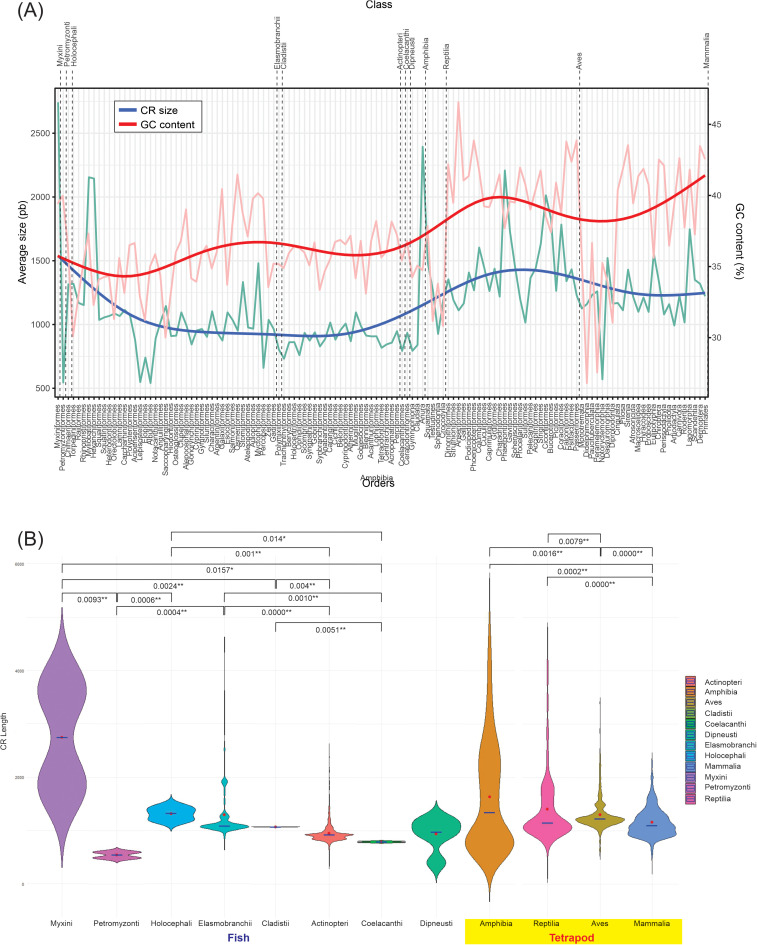
Variation in mitochondrial RC length in vertebrates. A. Control region length (bp) versus GC content. Blue and red lines show normalized CR length and GC content, respectively. Gray lines represent the mean length and GC content respectively in each taxonomic order. Dotted lines indicate clustering of taxonomic classes. B. Violin and box plots of CR length (bp) by vertebrate class, showing distribution patterns and mean values (indicated by black dots). Statistical differences between classes and between fish and tetrapods are shown (* p < 0.05; ** p < 0.01). Together, these results highlight pronounced evolutionary differences in CR length and composition among vertebrate lineages..

We have characterized and compared a total of 5,235 complete CRs available in NCBI’s GenBank representing 11 vertebrate taxonomic classes. The average CR length in vertebrates is 1,111.65 bp (± 405.5) and ranges from 329 bp in *Percopsis transmontana* (Actinopteri) to 4,912 bp in *Odorrana ishikawae* (Amphibia). Importantly, CR length variability within and among the studied taxonomic Classes was moderate to high (Fig 1A; S1 Table). CR length differed significant across vertebrate classes (Kruskal-Wallis, p < 2.2e-16). Post hoc Dunn’s test with Bonferroni correction identified significant pairwise differences (Z = −37.24, p < 0.001) between most classes (S2 Table). In particular, fishes generally exhibited shorter CRs than tetrapod classes, reflecting substantial lineage-specific expansions in terrestrial groups.

### 3.2. Variation in CR

#### 3.2.1. Fish lineages.

Of the 5,235 sequences analyzed, 2,861 CR (54.6%) belonged to fish; divided into 8 taxonomic classes (S1 Table). In fish, the average CR length was 969.25 (± 239.5) bp and ranged from 329 bp in *Percopsis transmontana* (Actinopteri; Percopsiformes) to 4,490 in *Aetobatus flagellum* (Elasmobranchii; Myliobatiformes). Fish clades with longer CRs generally exhibited greater length variability than those with shorter CRs. For example, Myxiniformes, Myliobatiformes, and Hexanchiformes had both long average CRs (2,743, 2,154.6, and 2,144.4 bp, respectively) and high variability (SD = ±1,251.5, ± 616.2, and ±887.8 bp, respectively). In contrast, Torpediniformes, Rajiformes, and Rhinopristiformes possessed shorter CRs (1,319, 1,169.3, and 1,152.3 bp, respectively) with lower variability (SD = ±10.1, ± 92.0, and ±226.0 bp, respectively). Several shark orders, including Squaliformes, Squatiniformes, Heterodontiformes, Lamniformes, and Carcharhiniformes, displayed intermediate levels of CR length variation. Overall, CR length variability tended to increase with CR expansion across fish lineages (Fig 1).

The CR in the Class Actinopteri had a mean length of 947.1 bp (± 187.5) with groups of early branching fish belonging to the Orders Acipenseriformes, Lepisosteiformes, Elopiformes, and Albuliformes having a slightly shorter CR (798.1 ± 176,1 bp) than teleostei fish (48 remaining orders) which have an average CR length equal to 949.1 ± 186.8 bp. Likewise, the CR in the classes Coelacanthi and Dipneusti had a mean length of 793 ± 16.9 and 934.6 ± 321.8 bp, respectively (see Fig 1A and S1 Table). Overall, CR length was different among all examined fish classes; Myxini, Petromyzonti, Holocephali, Elasmobranchii, Cladistii, Actinopteri, and Coelacanthi (Kruskal-Wallis, p < 0.001, post hoc Dunn’s test with Bonferroni correction, p < 0.001 in all paired comparisons) except Dipneusti against all other classes (Dunn’s test, p > 0.05 in all paired comparisons) (Fig 1B and S2 Table).

#### 3.2.2. Tetrapoda lineages.

A total of 2,374 CR sequences of tetrapods (45.34% of all sequences), distributed in 4 taxonomic classes were characterized. Our analysis shows that the average CR length in the Tetrapoda is 1,283.27 bp (SD ± 489.6) and ranged between 347 bp in *Tragulus napu* (Mammalia: Artiodactyla) to 4,704 bp in *Mantella madagascariensis* (Anura: Mantellidae) (Fig 1A and S1 Table).

Among tetrapods, the Class Amphibia had the longest average CRs and presented the greatest variation in CR length (1,633.2 ± 1023.53 bp) (Fig 1B). Within amphibians, anurans were characterized by long CRs (2,393.6 ± 843.6) while Gymnophiona and Caudata exhibited shorter CRs (934.6 ± 320.1 and 793.6 ± 392.6 bp, respectively). In Reptilia, CR length is 1404.3 ± 642.6 bp; with considerable variation among the orders analyzed (Fig 1A). Although, most of Aves CRs are relatively similar in length, CR length variability among bird orders was moderate (1,297.7 bp ± 292.5 bp) (See Fig 1A). Similarly, mammals also have relatively similar CRs in terms of length (1,158.08 ± 265.6 bp) (see S3 Table and Fig 1). A Kruskal-Wallis test found differences in the average length of the CR among tetrapods (χ²(3) = 131.6, p < 0.001) and an a posteriori test demonstrated that all classes were different (Dunn’s test, p < 0.001) other than amphibians vs. reptiles (Dunn’s test, p > 0.05). Overall, birds and mammals exhibited shorter CRs than the other tetrapod classes (Fig 1B and S2 Table).

### 3.3. CR duplication events in vertebrates

Among the 2,374 tetrapod mitochondrial control regions analyzed, 283 duplication events were detected, corresponding to an overall frequency of 11.9%. CR duplications were observed exclusively in non-mammalian tetrapods. Classes with the highest frequency of duplication were Reptiles (35.3% [108 out of 306 analyzed CRs]) and Aves (20.16% [158/767]) while CR duplications in Amphibia were less common (7.1% [17/241]). Furthermore, within each Class, CR duplications were restricted to specific taxonomic groups. For example, within reptiles, CR duplications were only detected in the order Squamata but the same were commonly observed in all species of the suborder Serpentes (detected in 37 of the 42 snakes analyzed) and in species belonging to the families Agamidae, Chamaelonidae and Varanidae. Similarly, within Aves, CR duplications were detected in most (72%) of the studied species of raptors (Order Accipitriformes, Falconiformes) as well as in Phoenicopteriformes and Piciformes (78.9%). CR duplications were less frequent in Passeriformes (23%), restricted to families such as Acanthizidae, Alaudidae, Calyptomenidae, Hirundinidae, Leiothrichidae, Petroicidae, Phylloscopidae, Pycnonotidae, Sylviidae, Zosteropidae and Ardeidae. In Amphibia, duplications were not common; sporadically present in some species belonging to the Anura and Caudata (S1 Table).

### 3.4. Nucleotide composition and strand asymmetry of vertebrate CRs

Vertebrate CRs exhibited low GC content (33.4% ± 4.3) and were characterized by near-zero AT-skew (0.0137 ± 0.0633) and strongly negative GC-skew values (−0.2356 ± 0.0988) (Fig 1A; S1 Table). Although GC content differed significantly among vertebrate groups (Kruskal–Wallis, p = 0.0017), post hoc analyses indicated that these differences were mainly driven by fishes, which showed significantly lower GC content than birds and mammals (p < 0.001), whereas amphibians, reptiles, and mammals did not differ significantly from one another (S2 Table).

#### 3.4.1. Fish lineages.

Fish CRs were generally AT-rich, with a mean GC content of 35.5 ± 3.1%. GC content varied substantially among orders, ranging from low values in Torpediniformes and Saccopharyngiformes (~30%) to higher values in Galaxiiformes and Osmeriformes (>40%) (S3 Table). Elasmobranchs exhibited the greatest GC-content variability, with values ranging from 28.1% in Arhynchobatidae to 38.1% in Dasyatidae (Fig 1). Significant differences in GC content were detected among the major fish classes (Kruskal–Wallis, p < 0.001), with Elasmobranchii showing significantly lower GC content than Petromyzonti, Cladistii, and Actinopteri (Dunn’s test, p ≤ 0.015; S2 Table). Most fish CRs exhibited slightly positive AT-skew values (0.019 ± 0.042) and negative GC-skew values (−0.183 ± 0.064), indicating a modest excess of A over T and a pronounced excess of C over G. However, Myxini, Petromyzonti, and several elasmobranch orders (Rajiformes, Rhinopristiformes, Squatiniformes, and Heterodontiformes) showed slightly negative AT-skew values (−0.09 to −0.01), reflecting a relative enrichment of T over A (S3 Table; S1 Fig; S2 Table).

GC-skew values exhibited a clear phylogenetic pattern across fishes, ranging from strongly negative values in Myxini (−0.453 ± 0.005) to progressively weaker biases in Petromyzonti, Holocephali, Elasmobranchii, Cladistii, and Actinopteri (−0.26 to −0.18). Within Actinopteri, GC-skew varied substantially, shifting from negative values in Acipenseriformes to near neutrality in Beryciformes and positive values in Perciformes. Likewise, sarcopterygian lineages (Coelacanthiformes and Dipneusti) exhibited positive GC-skews (~0.15), indicating a phylogenetically structured transition in strand compositional bias across ray-finned and lobe-finned fishes.

#### 3.4.2. Tetrapoda lineages.

In tetrapods, the CR exhibited a GC-content equal to 39.68 (±4.48) and ranged from 17.62% in *Petaurus breviceps* (Mammalia) to 50% in *Miniopterus fuliginosus* (Mammalia) (S1 Table). Considerable variability in GC-content was observed among taxonomic orders (Fig 1). For example, Didelphimorphia (Mammalia) exhibited the lowest GC-content (26.80 ± 2.84), while Anseriformes (Aves) exhibited a much greater GC-content (46.56 ± 1.28) (S3 Table). In addition, Amphibia and Reptilia had similar GC-content values (34.79 ± 3.28 and 35.58 ± 4.84%, respectively) while birds and mammals have relatively higher GC-content values (42.39 ± 3.28 and 40.02% ± 3.58%, respectively) (S1 Table). A Kruskal-Wallis test denoted differences in CR GC-content among the different tetrapod classes (χ²(3) = 823.73, p < 0.001) and a posteriori Dunn’s test indicated significant variation among all four compared classes (Amphibia, Reptilia, Aves and Mammalia, p < 0.001 in all paired comparisons) (S2 Table).

Tetrapod CRs showed near-neutral AT-skew values (0.007 ± 0.081) and strongly negative GC-skew values (−0.299 ± 0.096), indicating an overall excess of C relative to G. However, nucleotide composition varied among major clades. Amphibians, reptiles, and birds generally exhibited negative AT-skews, whereas mammals showed positive AT-skews (S1 Table). Considerable variation was also observed within classes; for example, Crocodylia displayed positive AT-skews in contrast to Squamata and Testudines. Similarly, birds exhibited an increase in AT-skew across lineages, from early-diverging orders to more derived groups such as Passeriformes, suggesting a directional shift in adenine enrichment relative to thymine. Mammals exhibited the greatest variation in AT-skew, with some basal lineages showing negative values, whereas most eutherian groups displayed positive AT-skews, reaching the highest values in Dermoptera and Chiroptera (S3 Table; S3 Fig). In contrast, GC-skew varied less among tetrapod classes and remained consistently negative across Amphibia, Reptilia, Aves, and Mammalia, indicating a general excess of C relative to G throughout tetrapod mitochondrial control regions (S3 Fig).

### 3.5. Tandem repeats in vertebrate CRs

Repetitive elements (REs) were identified in 43.2% (2,264/5,235) of the analyzed CRs, although their distribution varied substantially among vertebrate groups. REs were detected in 34.9% of fish CRs (1,000/2,861), where they ranged from 40 to 842 bp and comprised 1–14 tandem repeat units (mean = 1.84 ± 1.45 among repeat-containing CRs). These repeats were generally AT-rich (55–75% AT content). Fish exhibited fewer tandem repeats per CR on average (0.52) than tetrapods (1.22), and this difference was highly significant (Kruskal–Wallis, p < 0.001; Dunn’s test, p < 0.0001). In fish, REs was detected in all CRs of Myxini and Petromyzonti and in 50% of Holocephali, whereas the remaining classes showed lower frequencies (<50%). Significant differences in the number of tandem repeats were observed among fish classes (Kruskal–Wallis χ²(7) = 38.86, p < 0.001). Dunn’s post hoc test indicated that Elasmobranchii differed significant from Petromyzonti, Cladistii, and Actinopteri (p < 0.001), suggesting distinct evolutionary trends in mitochondrial organization.

Among tetrapods, 53.2% (1,264/2,374) of CRs contained REs, varying from 22 bp to over 1,600 bp and comprising 1–20 tandem units (mean = 2.27 ± 1.79). The AT content ranged from 56% to 100%, with no consistent nucleotide pattern. RE frequency differed across classes (Amphibia (57.2%), Reptilia (70.5%), Aves (42.1%), and Mammalia (56.4%)) and the number of tandem repeats varied significant (χ²(3) = 125.74, p < 0.001), with Mammalia exhibiting the highest counts.

**Fig 2:**
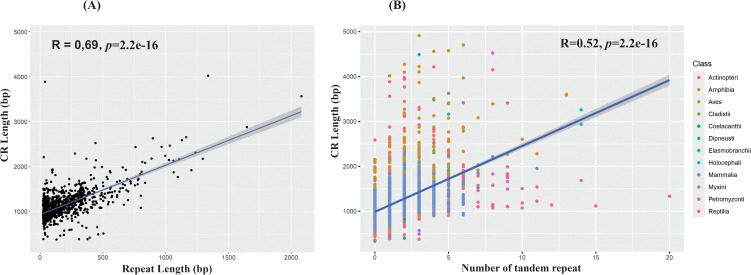
Correlation of control region length (bp) with repetitive element features detected across vertebrate mitogenomes. A. Correlation of control region length (bp) versus repeats length. Whole CR correlation in vertebrates against repeats length with a directly proportional relation and Pearson coefficient of 0,69 (p < 0.01). B. Correlation of control region length (bp) versus number of tandem repeats. Pearson coefficient of 0,52 (p < 0.01).

CR length was positively correlated with both the total length of tandem repeats (r = 0.69, p < 0.001) and the number of repeat units (r = 0.52, p < 0.001), indicating that repeat accumulation is a major contributor to CR expansion (Fig 2). In tetrapods, particularly long CRs (≥1,700 bp) were frequently associated with high repeat densities, notably in Anura and several avian orders, including Phaethontiformes, Gaviiformes, Ciconiiformes, and Bucerotiformes. In taxa with expanded CRs, including Anura and several avian orders, more than 90% of CRs contained tandem repeats. In contrast, short CRs (≤1,100 bp), particularly in many mammalian lineages such as Perissodactyla, Artiodactyla, Carnivora, Rodentia, and Primates, showed a low incidence of repeats (<30% of CRs; S1 Table). This pattern supports a strong association between CR expansion and repeat accumulation in tetrapods. Significant positive correlations between CR length and repeat number were observed in mammals (R = 0.37), amphibians (R = 0.40), and birds (R = 0.18), whereas no significant relationship was detected in reptiles (R = 0.06), suggesting greater structural constraint in reptilian CRs.

### 3.6. Conserved regulatory domains of the vertebrate CR

#### 3.6.1. Domain I: TAS/ETAS motifs (coreTAS Mix (M) and coreTAS LessStrict (LS)).

**Fig 3:**
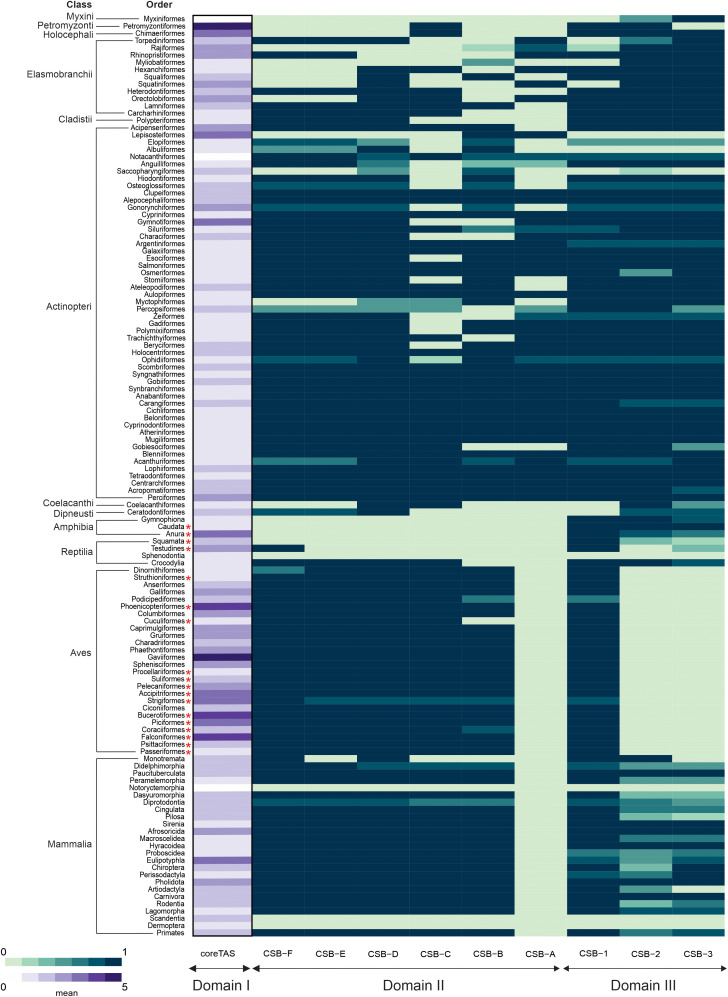
Heatmap of control region characterization. Characterization of Domains I, II and III. The blue color scale shows the mean number of coreTAS copies identified for each taxonomic order. The gray scale represents the relative frequency (ranging from 0 to 1) of domains I and II within the control region by order and classes. The red asterisk represents the presence of duplicate CRs in the taxonomical order.

The coreTAS motif was detected in 92.8% (4,860/5,235) of vertebrate CRs, with an average of 1.61 copies per sequence. However, copy number varied considerably, ranging from complete absence in 375 CRs to as many as 32 copies in *Enteromius trimaculatus* (Cyprinidae). Both the presence and abundance of coreTAS showed strong taxonomic dependence, with marked differences among major vertebrate lineages. The coreTAS motif is particularly enriched in tetrapods, especially in Mammalia and Aves, while it is significant less frequent or nearly absent in more basal lineages such as Myxini, Petromyzonti, and Chondrichthyes (e.g., Elasmobranchii and Holocephali) (Fig 3). Among tetrapods, coreTAS was detected in 98.3% of the studied CRs (2,334 out of 2,374), highlighting its near-universal presence in this clade. In contrast, among fish, the motif was identified in 88.3% of sequences (2,526 out of 2,861), with 11.7% lacking any coreTAS copies (S1 Table).

CoreTAS copy number differed significantly between major vertebrate groups. Tetrapods possessed more copies per CR on average (1.78 ± 1.42) than fishes (1.46 ± 1.75), and this difference was highly significant (Kruskal–Wallis, p < 0.0001; Dunn’s test, p < 0.0001). These results suggest greater retention or expansion of coreTAS motifs in tetrapods relative to fishes. Across vertebrates, CR length showed a weak but significant positive correlation with coreTAS copy number (R = 0.24, p < 0.0001; S3 Fig), indicating that longer CRs tend to contain more coreTAS motifs. This relationship was particularly strong in fishes (R = 0.52, p < 2.2 × 10 ⁻ ¹⁶), suggesting that coreTAS expansion contributes substantially to CR elongation in fish lineages. Among tetrapods, mammals (R = 0.37, p < 2.2e-16) and amphibians (R = 0.40, p < 6.4e-14) also exhibited moderate positive correlations between the examined variables, suggesting similar evolutionary trends. In contrast, birds showed a weaker correlation (R = 0.18, p = 7.4e-07), and no statistically significant relationship was found in reptiles (R = 0.06, p = 0.22) (S3 Fig and Fig 3). These findings indicate that CR length and coreTAS copy number often co-expand across vertebrates, but with marked lineage-specific variation. Furthermore, our analyses show a larger number of coreTAS copies in early branching fishes (except for Myxini with 0.5 copies ± 0.7071 copies) such as Petromyzonti, Holocephali and Elasmobranchii (4.5 ± 2.1213, 3.1 ± 0.9910 and 2.01 ± 1.0477 copies per CR, respectively) compared late branching clades; classes Actinopteri, Coelacanthi and Dipneusti, with copy number equal to 1.42 ± 1.7855, 1.0 ± 0, and 1.6 ± 0.5477, respectively (Fig 3).

CoreTAS copy number showed limited variation among major tetrapod classes but substantial heterogeneity within lineages. Amphibians exhibited the highest average copy number, driven largely by Anura, whereas Gymnophiona and Caudata typically retained a single copy. Among reptiles, turtles averaged slightly more than two copies, while Squamata, Sphenodontia, and Crocodylia generally contained fewer than 1.5 copies per CR. Birds displayed greater variation, with some orders (e.g., Phoenicopteriformes, Bucerotiformes, and Falconiformes) averaging about four copies, whereas most others contained one to three. Mammals were generally conserved at around two copies, although shrews (Soricidae) exhibited nearly four copies on average, while most primates retained a single copy (S3 Table; Fig 3). Additionally, coreTAS motifs were predominantly located in Domain I of fish CRs, whereas in tetrapods they occurred across both Domains I and II, indicating lineage-specific differences in motif organization (S4 Fig).

#### 3.6.2. Domain II: Central conserved sequence blocks (CSB-A to CSB-F).

Six conserved sequence blocks (CSB-A to CSB-F) were identified in the vertebrate CR, with notable variation among classes. No CSBs were detected in Myxini, and only CSB-C was detected in Petromyzonti and Holocephali. In contrast, in Elasmobranchii, CSB-D was detected in 74% of sequences, followed by moderate occurrences of CSB-F, CSB-E, CSB-C and CSB-A (~50%), while CSB-B was less often detected (23%). Actinopteri exhibited the highest overall CSBs presence, with frequencies exceeding 75% for all blocks except CSB-C, which was present in 65% of the CRs (see Fig 3).

In tetrapods, Domain II region typically includes a conserved block from CSB-F to CSB-B, with CSB-A consistently absent. However, Amphibia and most species of the Reptilia lacked all CSBs, with the exception of Testudines and Crocodylia, in which this region was commonly detected; 98% presence of CSB-F in Testudines and 100% presence of CSB-F to CSB-C in Crocodylia. In contrast, several mammalian orders, including Notoryctemorphia, Scandentia and Dermoptera lack CSB-F to CSB-B with only 30–60% of its length their CRs (S3 Table and Fig 3).

#### 3.6.3. Domain III: CSB1-CSB3 motifs.

Our analysis shows that domain III is the most conserved domain in vertebrates. In fish, the three CSBs (1–3) are present in more than 82% of the CRs analyzed, with some exceptions such as Myxiniformes (Myxini), Lepisosteiformes, Albuliformes and Saccopharyngiformes (Actinopteri), where these motifs were not detected. Normally, domain III in tetrapods is characterized by possessing a highly conserved CSB-1 domain (88% frequency), but with a low frequency of CSB-2 and 3 domains (both with 33%). The frequency of CSB-2 and CSB-3 domains varied among vertebrate classes (Fig 3). These domains were entirely absent in all analyzed sequences from Aves, whereas they were more commonly detected in Amphibia and Mammalia, with frequencies exceeding 82% and 60%, respectively.

### 3.7. Evolutionary relationships among CSB motifs

Maximum-likelihood analysis of class-level consensus motifs for CSB-A to CSB-F revealed varying levels of sequence conservation across vertebrate classes (S5 Fig). CSB-B was present in only four classes, all of which retained the TRCA motif at the 5′ end, except Elasmobranchii. At the 3′ end, distinct motifs were detected: a GGT motif in Aves and Mammalia, TAnnnCTCnTnA in Elasmobranchii, and a GCC motif in Actinopteri. Among all CSBs, CSB-C exhibited the highest sequence divergence across classes, as reflected by motif comparisons. Notably, phylogenetic structure of CSB-D motifs mirrored major evolutionary lineages, forming two well-supported clades: one consisting of tetrapods (represented by Mammalia and Aves) and the other comprising fish lineages, including Actinopteri, Elasmobranchii, Holocephali, Cladistii, and Dipneusti. Notably, Reptilia did not cluster with other tetrapods, instead grouping with basal vertebrate lineages, suggesting distinct evolutionary trajectories or motif turnover in this clade. The motif logos further support this structure, showing high conservation of CTGG and GC-rich motifs in tetrapods, in contrast to greater sequence variability and class-specific patterns among fish (S5F Fig). Likewise, CSB-E showed positional shifts in key motifs: a guanine triplet occurred at the 5′ end in Elasmobranchii, Holocephali, Cladistii, Actinopteri, and Dipneusti, but at the 3′ end in Aves, Mammalia, and Reptilia (S5G Fig). CSB motifs exhibited clear lineage-specific patterns. Amphibians, reptiles, and mammals shared a distinctive CAT motif in CSB-C, while CSB-F contained a conserved central KAAA motif with class-specific flanking sequences. Overall, motif composition across CSB-B to CSB-F consistently mirrored the major evolutionary split between fishes and tetrapods (S5 Fig).

## 4. Discussion

### 4.1. Large-scale comparative patterns of vertebrate CR evolution

In this study, we performed a large-scale comparative analysis of 5,235 vertebrate mitochondrial control regions (CRs), revealing extensive variation in CR length, nucleotide composition, repetitive content, and conserved regulatory domain organization across vertebrate lineages. These results challenge the traditional view of the CR as a broadly conserved region [4,25], and instead support a dynamic evolutionary model characterized by lineage-specific structural diversification. Tandem repeat accumulation emerged as a major driver of CR expansion, although repeat organization varied substantially among taxa. Patterns of CR duplication, motif turnover, and domain rearrangement further indicate independent trajectories of regulatory evolution among vertebrate groups [26,27].

### 4.2. Structural variation and expansion dynamics of vertebrate CRs

Our results reveal substantial variation in CR length across vertebrates, with tetrapods generally exhibiting longer and more variable CRs than fishes. In general, fish (including jawless, cartilaginous, and ray-finned lineages) possess significant shorter and less variable CRs, while tetrapods, particularly Amphibia and Reptilia, exhibit both longer and more variable CRs (Fig 1). Our results are in agreement with previous studies demonstrating high variability in the length of the CR in fish [15,28], in amphibians [29,30], in reptilian [8,13,16,31], avian [10,32] and mammals [4,7].

Our findings indicate that variation in vertebrate CR length is primarily driven by tandem repeat accumulation. A strong positive correlation was detected between CR length and both the length of repetitive regions (r = 0.69) and the number of tandem repeats (r = 0.52). Longer CRs (>1200 bp) typically contained extended repetitive segments (>230 bp) with multiple tandem repeats, whereas shorter CRs (<1100 bp) had fewer and smaller repeats. These results suggest that CR expansion throughout vertebrate evolution primarily reflects the progressive amplification of short repetitive sequences. Several mechanisms, including slipped-strand mispairing, unequal recombination, replication slippage, and transcription-associated mutagenesis, may contribute to the origin and amplification of repetitive elements within the CR [33–37]. However, the relative importance of these processes remains unclear and likely varies among vertebrate groups.

The pattern of CR expansion is particularly pronounced in tetrapods, which possess longer and more variable CRs than fishes. Among tetrapods, anurans exhibit the most extensive CR expansion, largely attributable to the accumulation of tandem repeats. This elongation is strongly associated with the presence of tandem repeats, averaging 3.1 copies per CR, where each RE spans approximately 1,087.7 bp. Notably, even a single copy of this RE accounts for 45.4% of the average CR length in anurans. Previous studies have highlighted the substantial contribution of tandemly repeated sequences to overall CR size in anurans [38,39]. One hypothesis is that selection has favored CR repeat expansion in anurans to enhance mitochondrial regulatory flexibility in response to their complex life histories, high metabolic plasticity, and substantial shifts in energy demands during development and across diverse environments [40]. These conditions may impose selective pressures on regulatory flexibility in mitochondrial function, with CR tandem repeats providing potential sites for modulation of replication and transcription initiation [4,7]. Additionally, the relatively relaxed mitochondrial mutation constraints in anurans, which show higher mtDNA substitution rates compared to other tetrapods [39], may facilitate the accumulation and retention of repetitive elements by reducing the purifying selection that limits CR expansion in other lineages.

A similar pattern is observed in reptiles, particularly in the family Lacertidae (order Squamata), where the CR averages 2,725.2 bp and contains large tandem repeats (averaging 866.2 bp) that occupy about 31.8% of its length. In Testudinidae turtles, the CR averages 2,074.7 bp and includes a higher number of tandem copies (average 3.1), though each repeat is relatively short (59 bp). These patterns highlight how CR expansion can result either from a few long repetitive elements or the accumulation of many short repeats, reflecting lineage-specific strategies in mitochondrial genome evolution [4,41]. Differences in reptilian CR repeat architecture may represent lineage-specific adaptations, with Lacertidae lizards evolving longer, repeat-rich CRs in association with high metabolic activity and thermoregulatory demands [42,43]. In contrast, Testudinidae turtles, which exhibit low metabolic rates, slow development, and long lifespans, may experience relaxed selective constraints on CR structure, allowing the accumulation of many short repeats through neutral processes or mild selection for basic regulatory function [7,8,16]. These contrasting strategies likely reflect lineage-specific responses to physiological and life-history demands, suggesting that both adaptive selection and neutral drift contribute to CR evolution in reptiles [8,41]. Similarly, Myliobatiformes exhibit expanded CRs containing multiple tandem repeats, further supporting the important role of repetitive elements in shaping fish mitochondrial genomes. These repeats may enhance mitochondrial regulatory flexibility in elasmobranchs, a group characterized by long lifespans, large body sizes, and relatively low metabolic rates [44,45]. In this context, tandem repeats may provide additional replication or transcription initiation sites, helping maintain mitochondrial efficiency under variable environmental conditions, particularly in species exposed to fluctuating oxygen levels and temperatures [45,46].

The greater CR length and variability observed in tetrapods relative to fishes may result from lineage-specific differences in mitochondrial genome architecture, replication dynamics, and life-history traits [33,36,37]. For instance, life-history traits such as longer generation times and smaller effective population sizes (Nₑ), common in many tetrapods, can reduce the efficacy of purifying selection [47,48]. This allows mildly deleterious or neutral insertions, such as tandem repeats, to persist and accumulate over time [49,50].

Our study also reveals a strong correlation between CR length and repeat content, highlighting RE accumulation as a key driver of CR expansion. While previous studies described repeats in isolated taxa [4,28], this is the first to quantify the pattern systematically across all major vertebrate classes. The correlation supports the view that REs contributes to mitogenomic architectural evolution, similar to the role of transposable elements and duplications in nuclear genome expansion [51,52].

### 4.3. CRs repeat variation

Our large-scale comparative analysis reveals a strong correlation between tandem repeat expansion and CR length across vertebrates, suggesting that repeat accumulation is a key mechanism shaping mitochondrial genome architecture. This finding extends earlier observations from specific taxa [4,28] and aligns with models of genome evolution where repetitive elements modulate regulatory regions [53,54]. Unlike the largely neutral role of repeats in nuclear genomes [52], mitochondrial repeats may be functionally co-opted to enhance replication and transcription in response to lineage-specific metabolic and life-history demands [55,56]. We find that basal vertebrate lineages, including jawless fishes (Myxini, Petromyzonti) and cartilaginous fishes (Holocephali), retain long CRs and exhibit high frequencies of REs, while more derived groups such as teleosts, birds, and mammals often possess shorter CRs with fewer repeats. This phylogenetic gradient may reflect a trend toward CR streamlining in derived lineages, potentially driven by selection for replication efficiency and genomic stability [57,58]. Conversely, in basal taxa with small effective population sizes and reduced purifying selection [47], mildly deleterious or functionally neutral repeats may persist and expand. Differences in replication dynamics, including increased slippage rates [33], relaxed polymerase fidelity [59], and the abundance of origin-associated motifs [4], further contribute to repeat proliferation in these groups. Altogether, these results challenge the view of the vertebrate CR as structurally conserved [3], highlighting its evolutionary plasticity and the complex interplay of phylogeny, molecular mechanisms, and selection in shaping mitochondrial genome organization.

In this sense, the high AT content observed in most tandem repeats suggests that compositional biases contribute substantially to CR expansion across vertebrates. However, nucleotide skews and base composition patterns in mitochondrial CRs should be interpreted cautiously because they may be influenced not only by biological processes but also by methodological artifacts. Replication asymmetry during mtDNA synthesis can generate strand-specific mutational pressures that favor the accumulation of A/T-rich sequences and produce characteristic AT- and GC-skew patterns, particularly in non-coding regulatory regions such as the CR [36]. In addition, repeat-rich regions are known to be challenging for short-read sequencing and genome assembly algorithms, potentially leading to inaccurate estimates of repeat copy number, repeat length, and local nucleotide composition [27,60]. Although the broad patterns detected here are consistent across thousands of independently generated mitogenomes, some extreme cases of CR expansion or compositional bias may partly reflect assembly difficulties associated with long tandem repeat arrays. Future studies based on long-read sequencing technologies will be valuable for validating repeat-rich CR assemblies and disentangling biological variation from potential technical artifacts.

### 4.4. Lineage-Specific duplication and remodeling of the CR

Our results show that mitochondrial CR duplications are largely restricted to tetrapods, with the highest frequencies observed in reptiles and birds, suggesting multiple independent duplication events during amniote evolution. In contrast, the low prevalence of duplications in amphibians and their absence in fishes indicate that CR duplication is a derived rather than ancestral vertebrate feature. Notably, some amphibians, such as *Hemisus marmoratus*, possess up to four CR copies, representing an exceptional level of duplication within the group. These partial copies retain a high sequence similarity (~70%) to the original region but span only 30–60% of its length, suggesting relatively recent duplication events followed by limited sequence divergence; supporting recent origin and subsequent reduction through the Tandem Duplication–Random Loss (TDRL) model, in which large duplicated blocks undergo asymmetric or incomplete deletion, leaving residual CR segments [55,61]. Such partial copies are often accompanied by rearrangements of adjacent mitochondrial genes [5], reinforcing the role of TDRL as a major driver of mitogenomic structural variation. Empirical evidence from reptiles illustrates this process: in some turtles (*Dermochelys coriacea* and *Pelochelys cantorii*), partial duplications include conserved regulatory motifs CSB2 and CSB3, likely resulting from TDRL or recombination events [62,63]. In turn, in marine snakes, duplication of an intermediate CR segment between TAS and CSB1 preserves functional motifs while omitting other domains [31], suggesting that structural reduction does not necessarily eliminate regulatory capacity.

Our analysis indicates that approximately 30% of the CR duplications detected (85/283) were complete copies, retaining >90% of the original CR length and high sequence identity (>80%). These complete duplications were particularly common in snakes, raptors, seabirds, and parrots, indicating repeated independent evolution of dual CRs across diverse vertebrate lineages. In snakes, complete duplications have been linked to the regulation of mitochondrial replication and transcription under high metabolic demand or fluctuating thermal environments [54]. In raptors and seabirds, the maintenance of both CRs has been hypothesized to support stable mitochondrial control in support of energetically costly lifestyles such as sustained flight and long-distance migration [26,55]. In parrots, strong purifying selection may preserve CR function across both copies as a strategy to sustain mitochondrial efficiency throughout their long lifespans [64,65]. CR duplication has also been associated with increased longevity in birds, possibly via elevated mtDNA copy number and reduced oxidative stress [66], and may provide replication flexibility during energetically demanding activities [67] or in thermal stress adaptation in ectotherms [68]. This pattern suggests that CR duplication may provide adaptive advantages across vertebrates, including lineages exposed to variable environmental and developmental pressures. A key question is why do some vertebrate lineages repeatedly retain CR duplications while others, often equally exposed to energetic or environmental challenges, do not? Possible explanations include lineage-specific constraints on mitochondrial genome architecture [1,69], differences in the mutational mechanisms that generate duplications [55,70], or alternative strategies for coping with metabolic stress that obviate the need for duplicated control regions [71]. Addressing this asymmetry will require broader taxonomic sampling and experimental tests of CR function across diverse vertebrate groups.

Overall, CR duplication appears to arise through a combination of neutral and selective processes, representing a potentially important driver of vertebrate mitogenome evolution and functional diversification.

### 4.5. Evolution of conserved regulatory domains in vertebrates

#### 4.5.1. TAS/ETAS motifs and replication-associated evolution.

The near-universal presence of the coreTAS motif (92.8% of species) underscores its fundamental role in vertebrate mitochondrial transcription termination and replication initiation [7,22]. Most vertebrates retain a single coreTAS copy (58%), consistent with strong functional conservation and stabilizing selection. Nevertheless, multiple copies are common in several lineages (21% with two copies and 9.4% with three or more), indicating considerable structural flexibility. This variation is lineage-specific, with high copy numbers in shrews (Soricidae) and some bird groups, whereas most primates retain a single motif. Notably, coreTAS is enriched in tetrapods (98.3% of mammals and birds retain at least one copy) but reduced or absent in several Actinopteri orders as Notacanthiformes, Siluriformes and Cypriniformes, suggesting lineage-specific divergence in mitochondrial replication architecture. The absence of coreTAS in several Actinopteri lineages likely reflects multiple, nonexclusive mechanisms. The high structural dynamism of teleost mitogenomes, including frequent gene rearrangements and CR duplications or deletions [15,72], conditions that may relax selective constraints on canonical regulatory motifs. Consequently, functions typically associated with coreTAS may be performed by lineage-specific, noncanonical motifs that are not readily detectable using consensus-based approaches [22,61]. Indeed, TAS-like elements with minimal sequence similarity have evolved independently in other vertebrate mitogenomes [3,10,64]. Alternatively, the loss or reduction of coreTAS may be tolerated in teleosts due to their distinctive mitochondrial physiology and flexible responses to diverse environmental conditions [15,73]. Such ecological and metabolic diversity could favor regulatory solutions decoupled from the TAS-dependent architecture typical of tetrapods, involving novel elements, shifts in the timing or localization of replication, or compensatory increases in mtDNA copy number and replication flexibility [74,75]. Therefore, the information above suggests that the apparent absence of coreTAS in Actinopteri does not necessarily represent functional loss but rather highlights the evolutionary plasticity of mitochondrial control region architecture.

Our results show that tetrapods harbor more copies of coreTAS in the mitochondrial CR than fish (1.78 vs. 1.46 on average), reflecting ~1.2-fold enrichment. Because TAS elements regulate the transition from replication termination to transcription initiation [7,53], additional copies may increase regulatory flexibility and robustness. This enrichment parallels the greater structural complexity of tetrapod CRs [3,4] and may have been favored by the metabolic demands of terrestrial life, including oxygen regulation, thermoregulation, and locomotion [76,77]. Independent TAS expansions in birds, amphibians, turtles, and eulipotyphlan mammals may reflect lineage-specific adaptations linked to ecological and life-history demands. coreTAS replication in the CR of vertebrates likely involves tandem duplication, replication slippage, or recombination [7,22]. While some duplicated motifs may enhance mitochondrial control in taxa with fluctuating metabolic demands [55,78]. Overall, the higher copy number of coreTAS in tetrapods underscores how control region architecture evolves in concert with vertebrate energetics and ecological diversification. Additionally, our analysis of coreTAS positioning within the CR (see S4 Fig) shows that while coreTAS is typically located in domain I, additional copies frequently occur near CSB-1, suggesting possible regulatory convergence or redundancy [22]. The spatial distribution of coreTAS motifs indicates that their evolution is governed by both functional constraint and lineage-specific structural diversification. Collectively, these findings highlight the evolutionary flexibility of mitochondrial regulatory elements and suggest that, although coreTAS is highly conserved in tetrapods, alternative regulatory mechanisms may have evolved in some fish lineages to maintain mitochondrial genome function.

#### 4.5.2. Central domain diversification and lineage-specific motifs.

Our comparative analysis of six conserved sequence blocks (CSB-A to CSB-F) within Domain II of vertebrate mitochondrial CRs reveals pronounced differences in frequency, distribution, and sequence conservation among major lineages. CSB-D (84.2%) and CSB-F (80.8%) are the most consistently conserved elements, especially in Testudines (98%) and Crocodylia (100%), where they retain core sequence motifs despite lineage-specific flanking variation. In contrast, CSB-A is the rarest element (35.1% overall), being completely absent in all tetrapods and from ancestral fish lineages such as Myxini, Petromyzonti, and Holocephali; while occurring with moderate frequency in roughly half of Elasmobranchii sequences and is most frequent in Actinopteri (~80%). Also, CSB-C shows intermediate frequency (69.8%) but substantial motif turnover among classes.

Our findings suggest that Domain II CSBs originated early in gnathostome evolution, with CSB-D and CSB-F remaining highly conserved. Fishes generally retain a broader CSB repertoire, whereas tetrapods show a reduced Domain II, except for Testudines and Crocodylia, which preserve the ancestral F-to-C block. First, loss through relaxed selective constraint could occur if their original roles in replication or transcription regulation were absorbed by adjacent CSBs or structural elements, reducing the efficacy of purifying selection and allowing motif degradation [8,14]. Second, functional replacement by alternative sequence elements—either other conserved CSBs (e.g., CSB-F, CSB-B) or noncanonical lineage-specific motifs—could preserve regulatory functions despite loss of the ancestral motif. Such functional convergence is well documented in vertebrate CRs, with Elasmobranchii and several reptile lineages exhibiting unique motif substitutions that maintain core regulatory activity [15,79]. Third, structural rearrangements and domain reorganization of the CR, common in vertebrates, may have displaced CSB-A and CSB-C from functional contexts. In many tetrapods, a streamlined F–B configuration in Domain II suggests large-scale deletions or inversions that rendered these motifs nonessential, leading to their rapid divergence and disappearance [3,66,70].

Overall, the distribution and conservation patterns of CSBs in Domain II indicate that while certain conserved sequences (particularly CSB-D and CSB-F) have been maintained under strong evolutionary constraint across vertebrates, others such as CSB-A and CSB-C have been evolutionarily dispensable in some clades—particularly tetrapods—due to redundancy, replacement, or structural reorganization of the control region. This highlights the dynamic nature of the CR as a genomic element shaped by both functional conservation and architectural plasticity.

#### 4.5.3. Conservation and remodeling of Domain III motifs.

Our large-scale analysis of vertebrate mitochondrial CRs exhibits significant structural conservation, particularly in Domain III (CSBs 1–3). In fish, these CSBs are present in the majority of sequences analyzed, with exceptions observed in certain basal or specialized lineages such as Myxini, Lepisosteiformes, Albuliformes, and Saccopharyngiformes [80]. This widespread conservation suggests a strong functional constraint, likely reflecting essential roles in transcription initiation and replication priming, as previously discussed by Jin et al. [60]. In contrast to fish, tetrapods display a more variable pattern of CSB retention. CSB-1 remains highly conserved (88%) across tetrapod lineages, whereas CSB-2 and CSB-3 are less consistently retained. Notably, these elements are completely absent in Aves and largely missing in most Reptilia, indicating lineage-specific losses or modifications [8,14,43]. However, Amphibia and Mammalia retain CSB-2 and CSB-3 at higher frequencies (>82% and 60%, respectively), suggesting selective retention in these groups [8]. The structural stability of Domain III in fish and its selective retention in certain tetrapods underscore its critical role in mitochondrial genome regulation.

The phylogenetic clustering of consensus CSB motifs (S5 Fig) further supports the existence of lineage-specific patterns of motif conservation and remodeling. However, because CSB motifs are short conserved sequence elements, they contain a limited number of phylogenetically informative characters. Consequently, motif-based phylogenetic analyses should be interpreted cautiously, as they may be influenced by homoplasy, sequence convergence, and substitution saturation. Therefore, these analyses are intended primarily to illustrate broad patterns of motif similarity and divergence among vertebrate lineages rather than to reconstruct organismal evolutionary relationships.

Overall, the strong conservation of CSB-1 and the variable retention of CSB-2 and CSB-3 underscore the dynamic evolution of the mitochondrial control region, where functional constraints coexist with lineage-specific diversification. These patterns suggest that mitochondrial regulatory regions maintain essential functions while accommodating adaptations to diverse ecological and life-history conditions [3].

## 5. Conclusion

In conclusion, our comparative analysis of 5,235 vertebrate mitochondrial demonstrates that the CR is a highly dynamic regulatory region shaped by both functional constraints and lineage-specific evolutionary processes. We identified extensive variation in CR length, nucleotide composition, repeat content, and the organization of conserved regulatory motifs across vertebrates. Tandem repeat accumulation emerged as a major driver of CR expansion, particularly in tetrapods, whereas birds and reptiles exhibited evidence of CR compaction through motif loss, reduced repeat content, and simplification of regulatory architecture. Despite these structural changes, the persistence of key motifs and the occurrence of motif redundancy and positional shifts suggest that essential regulatory functions are maintained through alternative architectural arrangements. Collectively, these findings highlight the non-neutral evolution of mitochondrial non-coding DNA and support the view that the CR functions as a flexible regulatory platform that has diversified alongside vertebrate evolutionary history.

## Supporting information

S1 Fig Boxplot bivariate of AT/GC skew by taxonomic classes in fish control region. (TIF)

S2 Fig Boxplot bivariate of AT/GC skew by taxonomic classes in in most of the vertebrate control region. (TIF)

S3 Fig Correlation between control region (CR) length and number of coreTAS copies. A modest but statistically significant positive correlation was detected (Pearson’s R = 0.24, 𝑝 < 0.0001).
(TIF)

S4 Fig Location of Domain I of vertebrate control region. (A) Indicates positions of coreTAS LS (less strict) and **(B)** indicates positions of coreTAS M (mix) of domain I per taxonomic class in vertebrate control region. Rose lines indicate mean values of position in base pair (bimodal data), and yellow lines indicate standard deviation of those values. Numbers in parenthesis indicate number of species that present coreTAS in each position.
(TIF)

S5 Fig Maximum-likelihood trees of Domain II CSBs at taxonomic class level in vertebrate control region. (A) Tree for CSB-1, (B) Tree for CSB-2, (C) Tree for CSB-3, (D) Tree for CSB-B, (E) Tree for CSB-C, (F) Tree for CSB-D, (G) Tree for CSB-E, (H) Tree for CSB-F. Numbers in branches indicate bootstrap repetitions.
(TIF)

S1 Table CR sequence characterization in vertebrata. Nucleotide composition, CR duplicate, Domine I (coreTAS), Domine II (CSBA-F), Domine III (CSB1–3) and Repetitive elements characterization by individual CR sequences is indicated.
(XLSX)

S2 Table Statistical Pairwise analysis of vertebrate CR sequence. A post hoc Dunn’s test with Bonferroni correction performed after Kruskal-Wallis analysis.
(DOCX)

S3 Table Molecular features and conserved structural elements in the CR of vertebrates. (DOCX)

S4 Table CR sequence of motifs used for the search in Domains II-III characterization in vertebrates. (XLSX)
