# Supplementary material for: Evolutionary patterns of the mitochondrial control region in vertebrates: A large-scale comparative analysis
Source: PLoS One. 2026 Jul 10;21(7):e0353555. doi: 10.1371/journal.pone.0353555 (PMC13353940; doi:10.1371/journal.pone.0353555)
Supplement: S3 Table — (DOCX) [file pone.0353555.s008.docx]

**Table S3**. Molecular features and conserved structural elements in the CR of vertebrates

| **Class** | **Order** | **N mit** | **Average size pb** | **%CG** | **AT skew** | **GC skew** | **Domine I (coreTAS)** | **Domine II (Central Domine)** | | | | | | **Domine III** | | |
| --- | --- | --- | --- | --- | --- | --- | --- | --- | --- | --- | --- | --- | --- | --- | --- | --- |
|  |  |  |  |  |  |  |  | CSB-F | CSB-E | CSB-D | CSB-C | CSB-B | CSB-A | CSB-1 | CSB-2 | CSB-3 |
| Myxini (2) | Myxiniformes | 2 | 2743,0 | 39,41 | -0,0941 | -0,4534 | 0.5 ± 0.71 | 0,00 | 0,00 | 0,00 | 0,00 | 0,00 | 0,00 | 0,00 | 0,50 | 1,00 |
| Petromyzonti (2) | Petromyzontiformes | 2 | 545,0 | 39,98 | -0,0120 | -0,2674 | 4.5 ± 2.12 | 0,00 | 0,00 | 0,00 | 1,00 | 0,00 | 0,00 | 1,00 | 1,00 | 0,00 |
| Holocephali (8) | Chimaeriformes | 8 | 1314,6 | 36,43 | 0,0289 | -0,1899 | 3.125 ± 0.99 | 0,00 | 0,00 | 0,00 | 1,00 | 0,00 | 0,00 | 1,00 | 0,88 | 1,00 |
| Elasmobranchii (164) | Torpediniformes | 3 | 1319,0 | 30,10 | 0,0803 | -0,2124 | 1.66 ± 1.15 | 1,00 | 1,00 | 1,00 | 0,00 | 0,00 | 1,00 | 0,00 | 0,67 | 1,00 |
|  | Rajiformes | 36 | 1169,3 | 32,82 | -0,0038 | -0,2307 | 2.3 ± 0.67 | 0,00 | 0,00 | 0,00 | 0,00 | 0,19 | 0,78 | 0,22 | 1,00 | 1,00 |
|  | Rhinopristiformes | 10 | 1152,3 | 35,18 | -0,0010 | -0,2815 | 2.3 ± 1.42 | 1,00 | 1,00 | 0,10 | 0,00 | 0,00 | 1,00 | 0,90 | 1,00 | 1,00 |
|  | Myliobatiformes | 22 | 2154,7 | 37,32 | 0,0406 | -0,2704 | 2,31 ± 1.36 | 0,00 | 0,00 | 0,00 | 0,00 | 0,50 | 0,00 | 0,00 | 1,00 | 1,00 |
|  | Hexanchiformes | 5 | 2144,4 | 32,30 | 0,0651 | -0,2587 | 3,8 ± 1.92 | 0,00 | 0,00 | 1,00 | 1,00 | 0,00 | 1,00 | 1,00 | 1,00 | 1,00 |
|  | Squaliformes | 9 | 1036,2 | 34,08 | 0,0046 | -0,2144 | 1.66 ± 0.5 | 0,00 | 0,00 | 1,00 | 0,00 | 0,89 | 0,00 | 0,89 | 0,89 | 0,89 |
|  | Squatiniformes | 5 | 1056,0 | 34,26 | -0,0717 | -0,2326 | 2.8 ± 0.45 | 0,00 | 0,00 | 1,00 | 0,00 | 0,00 | 1,00 | 0,00 | 1,00 | 1,00 |
|  | Heterodontiformes | 2 | 1067,5 | 34,33 | -0,0114 | -0,2442 | 1.5 ± 0.71 | 1,00 | 1,00 | 1,00 | 1,00 | 0,00 | 0,00 | 1,00 | 1,00 | 1,00 |
|  | Orectolobiformes | 9 | 1086,4 | 31,31 | 0,0356 | -0,2659 | 2.33 ± 0.87 | 0,00 | 0,00 | 1,00 | 1,00 | 0,00 | 1,00 | 0,89 | 1,00 | 1,00 |
|  | Lamniformes | 13 | 1066,4 | 35,60 | 0,0187 | -0,2172 | 1.61 ± 0.77 | 1,00 | 1,00 | 1,00 | 1,00 | 1,00 | 0,00 | 1,00 | 1,00 | 1,00 |
|  | Carcharhiniformes | 50 | 1114,1 | 33,14 | 0,0208 | -0,2106 | 1,52 ± 0.79 | 1,00 | 1,00 | 1,00 | 1,00 | 0,00 | 0,00 | 1,00 | 1,00 | 1,00 |
| Cladistii (11) | Polypteriformes | 11 | 1070,9 | 36,51 | 0,0229 | -0,2040 | 1.27 ± 0.47 | 1,00 | 1,00 | 1,00 | 0,00 | 0,00 | 0,00 | 1,00 | 1,00 | 1,00 |
| Actinopteri (2667) | Acipenseriformes | 28 | 878,1 | 36,65 | 0,0250 | -0,1974 | 2.78 ± 1.23 | 1,00 | 1,00 | 1,00 | 0,93 | 1,00 | 0,00 | 1,00 | 0,96 | 0,96 |
|  | Lepisosteiformes | 6 | 548,7 | 32,91 | 0,0270 | -0,1908 | 3.5 ± 1.05 | 0,00 | 0,00 | 0,00 | 0,00 | 1,00 | 1,00 | 0,00 | 0,00 | 0,00 |
|  | Elopiformes | 4 | 740,8 | 31,24 | 0,0291 | -0,1842 | 1.0 ± 0 | 0,75 | 0,75 | 0,50 | 0,00 | 0,75 | 0,00 | 0,50 | 0,50 | 0,50 |
|  | Albuliformes | 2 | 541,0 | 35,01 | 0,0312 | -0,1776 | 1.0 ± 0 | 0,50 | 0,50 | 1,00 | 0,00 | 1,00 | 0,00 | 0,00 | 0,00 | 0,00 |
|  | Notacanthiformes | 6 | 891,8 | 35,87 | 0,0333 | -0,1710 | 0 | 1,00 | 1,00 | 0,83 | 1,00 | 0,83 | 0,83 | 0,83 | 0,83 | 0,83 |
|  | Anguilliformes | 52 | 1025,6 | 34,37 | 0,0354 | -0,1644 | 1.26 ± 0.87 | 1,00 | 1,00 | 0,69 | 0,00 | 0,33 | 0,33 | 1,00 | 1,00 | 0,98 |
|  | Saccopharyngiformes | 4 | 1145,3 | 30,01 | 0,0375 | -0,1577 | 1.75 ± 2.06 | 0,00 | 0,00 | 0,50 | 0,00 | 0,75 | 0,00 | 0,00 | 0,25 | 0,00 |
|  | Hiodontiformes | 2 | 909,5 | 33,04 | 0,0396 | -0,1511 | 1 ± 0 | 1,00 | 1,00 | 1,00 | 0,00 | 1,00 | 0,00 | 1,00 | 1,00 | 1,00 |
|  | Osteoglossiformes | 20 | 912,4 | 36,00 | 0,0417 | -0,1445 | 1.95 ± 1.39 | 0,80 | 0,80 | 0,80 | 0,00 | 0,80 | 0,00 | 0,80 | 0,75 | 0,80 |
|  | Clupeiformes | 92 | 1094,9 | 36,81 | 0,0438 | -0,1379 | 2.07 ± 1.95 | 0,99 | 0,99 | 1,00 | 1,00 | 1,00 | 1,00 | 1,00 | 1,00 | 0,99 |
|  | Alepocephaliformes | 27 | 992,4 | 39,02 | 0,0459 | -0,1313 | 1.85 ± 0.86 | 1,00 | 1,00 | 1,00 | 1,00 | 1,00 | 1,00 | 0,96 | 1,00 | 1,00 |
|  | Gonorynchiformes | 7 | 843,7 | 34,17 | 0,0480 | -0,1247 | 2.42 ± 4.24 | 0,86 | 0,86 | 0,86 | 0,00 | 0,86 | 0,00 | 0,86 | 0,86 | 0,86 |
|  | Cypriniformes | 807 | 953,5 | 33,93 | 0,0500 | -0,1181 | 1.04 ± 1.44 | 1,00 | 1,00 | 1,00 | 0,87 | 1,00 | 1,00 | 0,96 | 0,99 | 1,00 |
|  | Gymnotiformes | 3 | 964,7 | 35,89 | 0,0521 | -0,1115 | 3 ± 0 | 1,00 | 1,00 | 1,00 | 0,00 | 0,00 | 1,00 | 1,00 | 1,00 | 1,00 |
|  | Siluriformes | 148 | 903,2 | 36,46 | 0,0542 | -0,1049 | 1.05 ± 1.5 | 0,99 | 0,99 | 0,99 | 1,00 | 0,64 | 0,80 | 0,79 | 0,97 | 0,97 |
|  | Characiformes | 42 | 1103,4 | 34,86 | 0,0563 | -0,0983 | 1.78 ± 2.8 | 0,98 | 0,98 | 1,00 | 0,02 | 0,00 | 1,00 | 1,00 | 0,98 | 1,00 |
|  | Argentiniformes | 7 | 936,1 | 36,06 | 0,0584 | -0,0917 | 1.28 ± 0.49 | 1,00 | 1,00 | 1,00 | 1,00 | 1,00 | 1,00 | 0,86 | 0,86 | 0,86 |
|  | Galaxiiformes | 7 | 874,1 | 40,45 | 0,0605 | -0,0851 | 1.0 ± 0.58 | 1,00 | 1,00 | 1,00 | 1,00 | 1,00 | 1,00 | 1,00 | 1,00 | 1,00 |
|  | Esociformes | 6 | 1095,0 | 33,72 | 0,0626 | -0,0785 | 1.0 ± 0 | 1,00 | 1,00 | 1,00 | 0,00 | 1,00 | 1,00 | 1,00 | 1,00 | 1,00 |
|  | Salmoniformes | 64 | 1034,7 | 37,89 | 0,0647 | -0,0719 | 0.98 ± 0.13 | 1,00 | 1,00 | 1,00 | 1,00 | 1,00 | 1,00 | 1,00 | 1,00 | 1,00 |
|  | Osmeriformes | 15 | 951,4 | 41,47 | 0,0668 | -0,0653 | 1.06 ± 0.26 | 1,00 | 1,00 | 1,00 | 1,00 | 1,00 | 1,00 | 1,00 | 0,47 | 1,00 |
|  | Stomiiformes | 2 | 1333,0 | 38,79 | 0,0689 | -0,0587 | 1.0 ± 0 | 1,00 | 1,00 | 1,00 | 0,00 | 1,00 | 0,00 | 1,00 | 1,00 | 1,00 |
|  | Ateleopodiformes | 2 | 976,5 | 37,12 | 0,0709 | -0,0521 | 2.0 ± 0 | 1,00 | 1,00 | 1,00 | 1,00 | 1,00 | 0,00 | 1,00 | 1,00 | 1,00 |
|  | Aulopiformes | 8 | 967,1 | 39,77 | 0,0730 | -0,0455 | 1.0 ± 0 | 1,00 | 1,00 | 1,00 | 0,88 | 0,88 | 0,88 | 0,88 | 0,88 | 0,88 |
|  | Myctophiformes | 7 | 1480,3 | 40,16 | 0,0751 | -0,0389 | 3.42 ± 6.87 | 0,00 | 0,00 | 0,43 | 0,43 | 1,00 | 0,00 | 1,00 | 1,00 | 1,00 |
|  | Percopsiformes | 2 | 660,0 | 39,79 | 0,0772 | -0,0323 | 1.5 ± 0.71 | 0,50 | 0,50 | 0,50 | 0,50 | 0,00 | 0,50 | 1,00 | 1,00 | 0,50 |
|  | Zeiformes | 6 | 1036,3 | 33,55 | 0,0793 | -0,0257 | 1.0 ± 0 | 1,00 | 1,00 | 1,00 | 0,00 | 0,00 | 0,83 | 0,83 | 0,83 | 0,83 |
|  | Gadiformes | 15 | 963,4 | 35,20 | 0,0814 | -0,0190 | 1.13 ± 0.35 | 0,87 | 0,87 | 1,00 | 0,00 | 1,00 | 1,00 | 1,00 | 1,00 | 1,00 |
|  | Polymixiiformes | 2 | 803,0 | 35,18 | 0,0835 | -0,0124 | 1.0 ± 0 | 1,00 | 1,00 | 1,00 | 0,00 | 1,00 | 1,00 | 1,00 | 1,00 | 1,00 |
|  | Trachichthyiformes | 3 | 870,3 | 36,52 | 0,0856 | -0,0058 | 1.0 ± 0 | 1,00 | 1,00 | 1,00 | 1,00 | 0,00 | 1,00 | 1,00 | 1,00 | 1,00 |
|  | Beryciformes | 16 | 861,0 | 35,94 | 0,0877 | 0,0008 | 2.06 ± 1.44 | 1,00 | 1,00 | 1,00 | 0,00 | 1,00 | 1,00 | 1,00 | 0,94 | 0,94 |
|  | Holocentriformes | 4 | 861,8 | 36,42 | 0,0898 | 0,0074 | 1.5 ± 0.58 | 1,00 | 1,00 | 1,00 | 1,00 | 1,00 | 1,00 | 1,00 | 1,00 | 1,00 |
|  | Ophidiiformes | 6 | 759,2 | 36,31 | 0,0918 | 0,0140 | 2.0 ± 0.41 | 0,83 | 0,83 | 1,00 | 0,17 | 1,00 | 0,83 | 0,83 | 0,83 | 0,83 |
|  | Scombriformes | 72 | 934,6 | 36,01 | 0,0939 | 0,0206 | 1.94 ± 2.44 | 0,99 | 0,99 | 0,99 | 0,99 | 0,99 | 0,99 | 0,96 | 0,94 | 0,92 |
|  | Syngnathiformes | 46 | 874,0 | 35,08 | 0,0960 | 0,0272 | 1.04 ± 0.58 | 0,96 | 0,96 | 0,98 | 0,98 | 0,98 | 0,98 | 0,96 | 0,96 | 0,98 |
|  | Gobiiformes | 119 | 938,4 | 36,70 | 0,0981 | 0,0338 | 1.61 ± 0.8 | 0,98 | 0,98 | 0,99 | 1,00 | 1,00 | 1,00 | 1,00 | 1,00 | 1,00 |
|  | Synbranchiformes | 8 | 828,0 | 33,36 | 0,1002 | 0,0404 | 1.37 ± 0.74 | 1,00 | 1,00 | 1,00 | 1,00 | 0,88 | 0,88 | 0,88 | 0,88 | 0,88 |
|  | Anabantiformes | 25 | 885,4 | 34,69 | 0,1023 | 0,0470 | 1.24 ± 0.78 | 1,00 | 1,00 | 1,00 | 1,00 | 1,00 | 1,00 | 1,00 | 1,00 | 1,00 |
|  | Carangiformes | 118 | 1021,6 | 35,31 | 0,1044 | 0,0536 | 1.8 ± 1.87 | 0,87 | 0,87 | 0,87 | 1,00 | 0,99 | 1,00 | 1,00 | 0,84 | 0,84 |
|  | Cichliformes | 90 | 880,9 | 36,73 | 0,1065 | 0,0602 | 1.12 ± 0.33 | 0,97 | 0,97 | 0,97 | 1,00 | 1,00 | 1,00 | 1,00 | 0,99 | 1,00 |
|  | Beloniformes | 32 | 956,4 | 36,88 | 0,1086 | 0,0668 | 1.32 ± 0.64 | 1,00 | 1,00 | 1,00 | 1,00 | 1,00 | 1,00 | 1,00 | 0,97 | 1,00 |
|  | Cyprinodontiformes | 63 | 1007,2 | 36,56 | 0,1107 | 0,0734 | 1.14 ± 0.43 | 1,00 | 1,00 | 1,00 | 1,00 | 1,00 | 1,00 | 0,98 | 0,98 | 1,00 |
|  | Atheriniformes | 18 | 868,9 | 37,17 | 0,1128 | 0,0800 | 1.38 ± 0.7 | 1,00 | 1,00 | 1,00 | 1,00 | 1,00 | 1,00 | 1,00 | 1,00 | 1,00 |
|  | Mugiliformes | 17 | 1094,9 | 34,17 | 0,1148 | 0,0866 | 1.29 ± 0.69 | 1,00 | 1,00 | 1,00 | 1,00 | 1,00 | 1,00 | 1,00 | 1,00 | 1,00 |
|  | Gobiesociformes | 2 | 986,0 | 36,81 | 0,1169 | 0,0932 | 1.0 ± 0 | 1,00 | 1,00 | 1,00 | 1,00 | 0,00 | 0,00 | 1,00 | 1,00 | 0,50 |
|  | Blenniiformes | 3 | 913,0 | 33,10 | 0,1190 | 0,0998 | 1.3 ± 0.58 | 1,00 | 1,00 | 1,00 | 1,00 | 1,00 | 1,00 | 1,00 | 1,00 | 1,00 |
|  | Acanthuriformes | 64 | 916,1 | 36,82 | 0,1211 | 0,1064 | 1.42 ± 1.37 | 0,66 | 0,66 | 0,97 | 0,97 | 0,80 | 1,00 | 0,84 | 0,78 | 0,89 |
|  | Lophiiformes | 24 | 907,6 | 38,20 | 0,1232 | 0,1130 | 1.7 ± 0.69 | 1,00 | 1,00 | 0,96 | 0,96 | 1,00 | 1,00 | 1,00 | 1,00 | 1,00 |
|  | Tetraodontiformes | 119 | 817,4 | 35,64 | 0,1253 | 0,1197 | 1.09 ± 0.75 | 1,00 | 1,00 | 1,00 | 1,00 | 1,00 | 1,00 | 1,00 | 0,98 | 0,95 |
|  | Centrarchiformes | 62 | 844,6 | 36,07 | 0,1274 | 0,1263 | 1.58 ± 0.53 | 0,98 | 0,98 | 1,00 | 1,00 | 1,00 | 1,00 | 1,00 | 1,00 | 1,00 |
|  | Acropomatiformes | 17 | 857,8 | 38,16 | 0,1295 | 0,1329 | 1.58 ± 0.51 | 1,00 | 1,00 | 1,00 | 1,00 | 1,00 | 1,00 | 0,88 | 0,88 | 0,82 |
|  | Perciformes | 346 | 950,8 | 36,41 | 0,1316 | 0,1395 | 2.15 ± 0.52 | 0,95 | 0,95 | 0,95 | 0,88 | 0,95 | 0,98 | 0,92 | 0,91 | 0,90 |
| Coelacanthi (2) | Coelacanthiformes | 2 | 793,0 | 35,43 | 0,1337 | 0,1461 | 1.0 ± 0 | 0,00 | 0,00 | 1,00 | 1,00 | 0,00 | 0,00 | 0,00 | 1,00 | 0,50 |
| Dipneusti (5) | Ceratodontiformes | 5 | 934,6 | 36,81 | 0,1357 | 0,1527 | 1.6 ± 0.55 | 0,80 | 0,80 | 1,00 | 0,00 | 0,00 | 0,00 | 0,00 | 0,80 | 0,80 |
| Amphibia (241) | Gymnophiona | 31 | 793,7 | 34,23 | 0,1378 | 0,1593 | 1.09 ± 0.68 | 0,00 | 0,00 | 0,00 | 0,00 | 0,00 | 0,00 | 0,97 | 0,97 | 0,77 |
|  | Caudata | 86 | 839,5 | 35,07 | 0,1399 | 0,1659 | 1.03 ± 0.5 | 0,00 | 0,00 | 0,00 | 0,00 | 0,00 | 0,00 | 0,99 | 0,98 | 0,99 |
|  | Anura | 124 | 2393,6 | 34,74 | 0,1420 | 0,1725 | 3.10 ± 3.4 | 0,00 | 0,00 | 0,00 | 0,00 | 0,00 | 0,00 | 0,94 | 0,73 | 0,70 |
| Reptilia (306) | Squamata | 202 | 1484,3 | 37,85 | 0,1441 | 0,1791 | 1.53 ± 1.05 | 0,00 | 0,00 | 0,00 | 0,00 | 0,00 | 0,00 | 0,89 | 0,32 | 0,25 |
|  | Testudines | 85 | 1264,3 | 31,17 | 0,1462 | 0,1857 | 2.22 ± 1.22 | 0,98 | 0,00 | 0,00 | 0,00 | 0,00 | 0,00 | 0,98 | 0,11 | 0,33 |
|  | Sphenodontia | 1 | 926,0 | 33,80 | 0,1483 | 0,1923 | 1 | 0,00 | 0,00 | 0,00 | 0,00 | 0,00 | 0,00 | 0,00 | 0,00 | 0,00 |
|  | Crocodylia | 18 | 1190,0 | 30,82 | 0,1504 | 0,1989 | 1.11 ± 0.32 | 1,00 | 1,00 | 1,00 | 1,00 | 0,00 | 0,00 | 0,94 | 1,00 | 0,83 |
| Aves (767) | Dinornithiformes | 3 | 1354,3 | 42,19 | 0,1525 | 0,2055 | 1.33 ± 0.58 | 0,67 | 1,00 | 1,00 | 1,00 | 1,00 | 0,00 | 1,00 | 0,00 | 0,00 |
|  | Struthioniformes | 11 | 1190,4 | 39,48 | 0,1546 | 0,2121 | 1.36 ± 0.67 | 1,00 | 1,00 | 1,00 | 1,00 | 1,00 | 0,00 | 1,00 | 0,00 | 0,00 |
|  | Anseriformes | 32 | 1110,8 | 46,56 | 0,1567 | 0,2187 | 1.65 ± 0.83 | 1,00 | 1,00 | 1,00 | 0,97 | 1,00 | 0,00 | 0,97 | 0,00 | 0,00 |
|  | Galliformes | 67 | 1165,2 | 41,06 | 0,1587 | 0,2253 | 2.22 ± 1 | 1,00 | 1,00 | 1,00 | 0,99 | 1,00 | 0,00 | 1,00 | 0,00 | 0,00 |
|  | Podicipediformes | 3 | 1407,7 | 41,36 | 0,1608 | 0,2319 | 1.66 ± 1.15 | 1,00 | 1,00 | 1,00 | 1,00 | 0,67 | 0,00 | 0,67 | 0,00 | 0,00 |
|  | Phoenicopteriformes | 2 | 1268,5 | 43,87 | 0,1629 | 0,2385 | 4 ± 0 | 1,00 | 1,00 | 1,00 | 1,00 | 1,00 | 0,00 | 1,00 | 0,00 | 0,00 |
|  | Columbiformes | 24 | 1603,3 | 41,82 | 0,1650 | 0,2451 | 2.54 ± 0.88 | 1,00 | 1,00 | 1,00 | 1,00 | 0,92 | 0,00 | 1,00 | 0,00 | 0,00 |
|  | Cuculiformes | 4 | 1442,8 | 39,23 | 0,1671 | 0,2517 | 1.25 ± 0.5 | 1,00 | 1,00 | 1,00 | 1,00 | 0,00 | 0,00 | 1,00 | 0,00 | 0,00 |
|  | Caprimulgiformes | 20 | 1262,3 | 39,18 | 0,1692 | 0,2584 | 2.4 ± 1.14 | 1,00 | 1,00 | 1,00 | 1,00 | 0,90 | 0,00 | 0,95 | 0,00 | 0,00 |
|  | Gruiformes | 39 | 1438,1 | 40,17 | 0,1713 | 0,2650 | 2.74 ± 1.87 | 1,00 | 1,00 | 1,00 | 1,00 | 1,00 | 0,00 | 1,00 | 0,00 | 0,00 |
|  | Charadriiformes | 46 | 1218,7 | 41,48 | 0,1734 | 0,2716 | 1.93 ± 0.8 | 1,00 | 1,00 | 1,00 | 1,00 | 0,98 | 0,00 | 0,98 | 0,00 | 0,00 |
|  | Phaethontiformes | 2 | 2208,0 | 37,70 | 0,1755 | 0,2782 | 2.5 ± 0.71 | 1,00 | 1,00 | 1,00 | 1,00 | 1,00 | 0,00 | 1,00 | 0,00 | 0,00 |
|  | Gaviiformes | 2 | 1729,5 | 39,59 | 0,1776 | 0,2848 | 5 ± 0 | 1,00 | 1,00 | 1,00 | 1,00 | 1,00 | 0,00 | 1,00 | 0,00 | 0,00 |
|  | Sphenisciformes | 10 | 1449,0 | 39,50 | 0,1796 | 0,2914 | 2.2 ± 0.92 | 1,00 | 1,00 | 1,00 | 1,00 | 0,90 | 0,00 | 0,90 | 0,00 | 0,00 |
|  | Procellariiformes | 8 | 1197,1 | 41,96 | 0,1817 | 0,2980 | 1.12 ± 0.83 | 1,00 | 1,00 | 1,00 | 1,00 | 0,88 | 0,00 | 0,88 | 0,00 | 0,00 |
|  | Suliformes | 2 | 1014,5 | 40,80 | 0,1838 | 0,3046 | 2 ± 1 | 1,00 | 1,00 | 1,00 | 1,00 | 1,00 | 0,00 | 1,00 | 0,00 | 0,00 |
|  | Pelecaniformes | 26 | 1365,7 | 39,01 | 0,1859 | 0,3112 | 2.57 ± 1.03 | 1,00 | 1,00 | 1,00 | 1,00 | 1,00 | 0,00 | 1,00 | 0,00 | 0,00 |
|  | Accipitriformes | 23 | 1440,9 | 40,39 | 0,1880 | 0,3178 | 2.91 ± 1.31 | 1,00 | 1,00 | 1,00 | 1,00 | 0,96 | 0,00 | 1,00 | 0,00 | 0,00 |
|  | Strigiformes | 13 | 1632,7 | 41,77 | 0,1901 | 0,3244 | 3.15 ± 1.99 | 1,00 | 0,77 | 0,77 | 0,77 | 0,77 | 0,00 | 0,77 | 0,00 | 0,00 |
|  | Ciconiiformes | 3 | 2012,7 | 38,52 | 0,1922 | 0,3310 | 2 ± 0 | 1,00 | 1,00 | 1,00 | 1,00 | 1,00 | 0,00 | 1,00 | 0,00 | 0,00 |
|  | Bucerotiformes | 8 | 1825,6 | 37,41 | 0,1943 | 0,3376 | 3.62 ± 2.2 | 1,00 | 1,00 | 1,00 | 1,00 | 1,00 | 0,00 | 1,00 | 0,00 | 0,00 |
|  | Piciformes | 17 | 1264,2 | 40,90 | 0,1964 | 0,3442 | 3.17 ± 1.74 | 1,00 | 1,00 | 1,00 | 1,00 | 1,00 | 0,00 | 1,00 | 0,00 | 0,00 |
|  | Coraciiformes | 9 | 1783,9 | 38,72 | 0,1985 | 0,3508 | 1.66 ± 0.71 | 1,00 | 1,00 | 1,00 | 1,00 | 0,78 | 0,00 | 1,00 | 0,00 | 0,00 |
|  | Falconiformes | 13 | 1339,6 | 43,79 | 0,2006 | 0,3574 | 3.76 ± 0.6 | 1,00 | 1,00 | 1,00 | 1,00 | 1,00 | 0,00 | 0,92 | 0,00 | 0,00 |
|  | Psittaciformes | 51 | 1432,8 | 42,37 | 0,2026 | 0,3640 | 1.64 ± 0.8 | 1,00 | 1,00 | 1,00 | 1,00 | 1,00 | 0,00 | 1,00 | 0,00 | 0,00 |
|  | Passeriformes | 329 | 1226,9 | 43,88 | 0,2047 | 0,3706 | 1.22 ± 0.65 | 0,97 | 0,99 | 0,97 | 0,98 | 0,98 | 0,00 | 0,97 | 0,00 | 0,00 |
| Mammalia (1059) | Monotremata | 3 | 1125,7 | 35,65 | -0.095 | -0.087 | 2 ± 0 | 1,00 | 0,00 | 1,00 | 0,00 | 0,00 | 0,00 | 1,00 | 1,00 | 0,00 |
|  | Didelphimorphia | 4 | 1161,0 | 26,80 | 0.039 | -0.165 | 1.5 ± 1 | 1,00 | 1,00 | 0,75 | 0,75 | 0,75 | 0,00 | 0,75 | 0,50 | 0,50 |
|  | Paucituberculata | 2 | 1232,0 | 36,66 | -0,025 | -0.177 | 2 ± 1 | 1,00 | 1,00 | 1,00 | 1,00 | 1,00 | 0,00 | 1,00 | 1,00 | 1,00 |
|  | Peramelemorphia | 2 | 1260,0 | 27,55 | 0,2131 | 0,3971 | 1 ± 0 | 1,00 | 1,00 | 1,00 | 1,00 | 1,00 | 0,00 | 1,00 | 0,50 | 0,50 |
|  | Notoryctemorphia | 1 | 570,0 | 35,26 | 0,2152 | 0,4037 | 0 | 0,00 | 0,00 | 0,00 | 0,00 | 0,00 | 0,00 | 0,00 | 0,00 | 0,00 |
|  | Dasyuromorphia | 17 | 1521,5 | 33,94 | 0,2173 | 0,4103 | 1.88 ± 1.22 | 1,00 | 1,00 | 0,94 | 1,00 | 1,00 | 0,00 | 1,00 | 0,35 | 0,35 |
|  | Diprotodontia | 13 | 1229,9 | 31,00 | 0,2194 | 0,4169 | 1.61 ± 0.65 | 0,85 | 0,85 | 0,77 | 0,69 | 0,62 | 0,00 | 0,77 | 0,69 | 0,54 |
|  | Cingulata | 13 | 1168,8 | 40,38 | 0,2215 | 0,4235 | 1.61 ± 0.65 | 1,00 | 1,00 | 1,00 | 1,00 | 1,00 | 0,00 | 1,00 | 0,69 | 0,69 |
|  | Pilosa | 11 | 1111,5 | 41,86 | 0,2235 | 0,4301 | 1.81 ± 0.75 | 1,00 | 1,00 | 1,00 | 1,00 | 1,00 | 0,00 | 1,00 | 0,36 | 0,27 |
|  | Sirenia | 3 | 1430,0 | 43,54 | 0,2256 | 0,4367 | 1 ± 0 | 1,00 | 1,00 | 1,00 | 1,00 | 1,00 | 0,00 | 1,00 | 1,00 | 1,00 |
|  | Afrosoricida | 2 | 1216,0 | 39,45 | 0,2277 | 0,4433 | 2.5 ± 0.75 | 1,00 | 1,00 | 1,00 | 1,00 | 1,00 | 0,00 | 1,00 | 1,00 | 1,00 |
|  | Macroscelidea | 3 | 1099,7 | 41,36 | 0,2298 | 0,4499 | 1 ± 0 | 1,00 | 1,00 | 1,00 | 1,00 | 1,00 | 0,00 | 1,00 | 0,67 | 0,67 |
|  | Hyracoidea | 2 | 1211,5 | 41,87 | 0,2319 | 0,4565 | 1 ± 0 | 1,00 | 1,00 | 1,00 | 1,00 | 1,00 | 0,00 | 1,00 | 1,00 | 1,00 |
|  | Proboscidea | 7 | 1098,9 | 40,76 | 0,2340 | 0,4631 | 1 ± 0 | 1,00 | 1,00 | 1,00 | 1,00 | 1,00 | 0,00 | 0,71 | 0,57 | 0,71 |
|  | Eulipotyphla | 40 | 1558,5 | 35,61 | 0,2361 | 0,4697 | 3.17 ± 1. | 1,00 | 1,00 | 1,00 | 1,00 | 1,00 | 0,00 | 0,75 | 0,55 | 0,83 |
|  | Chiroptera | 103 | 1337,9 | 42,52 | 0,2382 | 0,4763 | 1.99 ± 2.01 | 1,00 | 1,00 | 1,00 | 1,00 | 1,00 | 0,00 | 0,94 | 0,30 | 0,87 |
|  | Perissodactyla | 15 | 1079,1 | 42,11 | 0,2403 | 0,4829 | 1.13 ± 0.52 | 1,00 | 1,00 | 1,00 | 1,00 | 1,00 | 0,00 | 0,73 | 0,67 | 1,00 |
|  | Pholidota | 8 | 1159,1 | 36,47 | 0,2424 | 0,4895 | 2.25 ± 1.58 | 1,00 | 1,00 | 1,00 | 1,00 | 1,00 | 0,00 | 1,00 | 1,00 | 1,00 |
|  | Artiodactyla | 242 | 992,2 | 39,43 | 0,2445 | 0,4961 | 1.58 ± 0.96 | 1,00 | 1,00 | 1,00 | 0,99 | 0,99 | 0,00 | 0,98 | 0,43 | 0,06 |
|  | Carnivora | 131 | 1226,0 | 42,62 | 0,2465 | 0,5027 | 1.51 ± 0.24 | 0,97 | 0,97 | 0,98 | 0,98 | 1,00 | 0,00 | 0,99 | 0,92 | 0,94 |
|  | Rodentia | 228 | 1015,9 | 37,75 | 0,2486 | 0,5093 | 1.80 ± 0.24 | 1,00 | 1,00 | 1,00 | 1,00 | 0,95 | 0,00 | 0,92 | 0,39 | 0,67 |
|  | Lagomorpha | 19 | 1746,5 | 41,82 | 0,2507 | 0,5159 | 1.42 ± 0.25 | 1,00 | 1,00 | 1,00 | 1,00 | 1,00 | 0,00 | 1,00 | 0,84 | 0,84 |
|  | Scandentia | 1 | 1350,0 | 37,26 | 0,2528 | 0,5225 | 2 | 0,00 | 0,00 | 0,00 | 0,00 | 0,00 | 0,00 | 0,00 | 0,00 | 0,00 |
|  | Dermoptera | 1 | 1320,0 | 43,48 | 0,2549 | 0,5291 | 1 | 0,00 | 0,00 | 0,00 | 0,00 | 0,00 | 0,00 | 0,00 | 0,00 | 0,00 |
|  | Primates | 188 | 1221,4 | 42,55 | 0,2570 | 0,5358 | 1.50 ± 0.26 | 0,99 | 0,99 | 1,00 | 1,00 | 1,00 | 0,00 | 0,98 | 0,70 | 0,80 |
