# Supplementary material for: Evolutionary patterns of the mitochondrial control region in vertebrates: A large-scale comparative analysis
Source: PLoS One. 2026 Jul 10;21(7):e0353555. doi: 10.1371/journal.pone.0353555 (PMC13353940; doi:10.1371/journal.pone.0353555)
Supplement: Attachment — Submitted filename: Comments to PONE-D-25-58380.pdf [file pone.0353555.s010.pdf]

Comments to the Editor:

The manuscript (Manuscript PONE-D-25-58380): "**The evolution of the control region in the mitochondrial genome of vertebrates**" addresses an important topic—the evolutionary dynamics of the mitochondrial control region (CR) across vertebrates—using a large dataset of 5,235 complete CR sequences spanning 12 classes. This scale exceeds many prior taxon-specific or smaller-scale analyses, and the focus on repetitive elements, conserved motifs (ETAS/TAS, CSBs), nucleotide composition, length variation, and duplications is relevant to mitogenome evolution, replication/transcription regulation, and lineage-specific diversification. The assembly and analysis of thousands of vertebrate CRs is ambitious and provides a valuable broad phylogenetic framework. The identification of lineage-specific patterns (e.g., longer/more variable CRs in tetrapods, especially amphibians and reptiles, vs. fishes; CR duplications largely restricted to certain reptiles and birds; correlations between CR length and tandem repeat abundance) offers useful empirical insights. The discussion of mechanisms like slipped-strand mispairing, replication slippage, and life-history influences on repeat accumulation is conceptually sound.

However, the manuscript has substantial issues that undermine its current suitability for publication in PLOS ONE. These concern methods transparency, reproducibility, data handling and potential errors, analytical rigor, structure and writing, and presentation of results. Many sections read as an early draft with inconsistencies, incomplete descriptions. Overall, the core idea and dataset have strong potential for PLOS ONE, which values large-scale comparative studies with broad relevance. However, major revisions are essential to elevate methodological rigor, fix errors, enhance transparency/reproducibility, and polish the presentation. I recommend resubmission after these issues are comprehensively addressed; a revised version could make a solid contribution to the field.

Comments to authors:

Main comments

- I would congratulate to authors for performing such a broad study with thousands of CRs sequences. I believe the manuscript will benefit much from the provided comments and well fit for the publication after revision. The provided text lacks a dedicated, detailed Methods section. Key elements are missing or only referred to: exact search strategy and filters used in GenBank/NCBI to retrieve the 5,235 "complete" CR sequences; criteria for defining "complete" CRs (critical given known assembly difficulties with repeats); bioinformatics pipeline for extraction, annotation of TAS/ETAS, central domain (CSB-A to F), and Domain III (CSB1-3); tools and parameters for motif discovery, consensus sequence generation, maximum-likelihood motif analysis (S5 Fig.), repeat detection (e.g., tandem repeat finder settings), statistical tests (Kruskal-Wallis, Dunn's with Bonferroni), and correlation analyses.
- How were partial or ambiguous CRs excluded? How were duplicates or heteroplasmy handled? Describe quality filtering, multiple sequence alignment (if used), and any custom scripts for domain identification. Without this, results are not reproducible.
- The manuscript's primary strength is its ambitious scale—analyzing over 5,000 vertebrate CR sequences—which enables robust documentation of inter- and intra-class variation in length, repeat content, nucleotide composition, and conserved motif copy number/position. This provides a valuable empirical foundation for understanding how tandem repeat proliferation drives CR expansion in tetrapods while core regulatory elements (TAS, CSBs) show differential conservation, revealing lineage-specific evolutionary trajectories (e.g., elongation in anurans/reptiles, streamlining in birds/mammals). When methods are fully detailed, this work could serve as a useful reference for mitogenomic researchers. Therefore, major weaknesses include insufficient methodological transparency (no detailed pipeline for sequence retrieval, CR annotation, or motif detection), numerous numerical inconsistencies and typographical errors suggesting the manuscript is not yet polished, and limited reproducibility due to incomplete data sharing. Analytical

descriptions for custom motifs and phylogenetic motif analyses need expansion, the novelty claim requires better contextualization against existing literature, and the text contains structural/grammatical issues. These issues currently compromise rigor and clarity.

Require thorough proofreading and verification:

- Abstract: "5,235 complete vertebrate CRs spanning 12 classes"; "tetrapods (1,283 ± 239.5 bp) than in fishes (969 ± 489.6 bp)". Later text reverses or varies SDs (e.g., fishes 969.25 ± 239.5; tetrapods 1,283.27 ± 489.6). Fish vs. tetrapod numbers shift slightly across pages.
- "11 vertebrate taxonomic classes" in one place, "12" in another; "8 taxonomic classes" for fishes.
- Minimum length 329 bp in *Percopsis transmontana* (note spelling: *transmontana*).
- Duplication frequencies: Reptiles 35.3% (108/306), Aves 20.16% (158/767), Amphibia 7.1% (17/241)—verify totals against overall 2,374 tetrapod sequences.
- Check the correct spelling of the scientific name and pay attention to the inconsistent capitalization and italics for taxa.

Analytical rigor and interpretation:

- Phylogenetic context for motif evolution (S5 Fig., ML analysis of consensus motifs) is promising but requires clearer methods (alignment, model selection, tree inference) and discussion of limitations (e.g., short motifs, saturation).
- Nucleotide skews and compositional biases: Discuss potential artifacts from replication asymmetry or sequencing/assembly biases in repeat-rich regions.
- Duplications: Clarify detection method and functional implications (e.g., concerted evolution in snakes/birds).
- Strengthen the "why these matters" section: Link findings more explicitly to broader questions in mitochondrial evolution, compensatory evolution, or implications for phylogenomics/barcoding (CR is often excluded due to variability).

Writing and presentation:

- **Title** is clear but could specify "large-scale comparative analysis" or similar for precision. Claims of "first comprehensive comparative framework" need tempering—while the scale is large, prior comparative mitogenomic work exists (including on CR in specific clades or broader vertebrate mitogenomes). Position the study more accurately as a large-scale synthesis rather than wholly unprecedented.
- Please write all the terms in full form when they appear at the first time; e. g ETAS: Extended Termination-Associated Sequences in Abstract
- The provided text is too lengthy, fragmented with repetitive phrasing or long sentences. Please improve its fluency, use concise scientific English and avoid redundancy.

- Provide a complete, standalone 'Methods section' with all bioinformatics details, parameters, and validation steps. Deposit data/code publicly and update the Data Availability statement with specific links/DOIs.

- Systematically verify and standardize all numerical results, and statistical reporting across abstract, results, tables, and figures. Include sample sizes (n) for all means ± SD and post-hoc tests. Several times '±' is missing when average (mean)±SD are reported. E.g. **Page 9, Line 57**: 'between 1,028 900 to 1,581 bp in Aves'/ **Page 22, Line 345:** and 2.01 1.0477 copies per…

- Please be consistent with format when reporting standard deviation: use +/- OR ± throughout the text; e.g. **Page 12, Line 122:** CR length was 969.25 (+/- 239.5) bp/ replace Fig 1a ----> Fig 1A

- Proofread thoroughly for grammar, typos, consistency (e.g., GC- vs. CG-, CSB- vs. CBS, fish vs. tetrapod stats), and flow. Consider professional editing if needed. E.g. **Page 15, Line 193**: statistically significant lower GC content...; replace 'significantly' with 'significant'/ **Page 17, Line 239**: delete 'the' a posteriori… and I would reword it as follow: "… *and a posteriori Dunn's test indicated significant variation among all four compared classes..*"

- Page 18, Line 250: Testudines

- Page 19, Line 290: the CR length was moderately correlated …

- Page 23, Line 372: frequently detected (74%)…

- Page 24, Line 381: Delete "in", also "ing": ...are lacking CSB-F to CSB-B within their CRs.

- Page 32, Line 579: These partial copies retain an important? Please check the sentence for its structure and meaning.

- Ensure all figures/tables are complete, high-resolution, and self-explanatory with proper legends! In current version, provided figures are in low quality I cannot read the words.

- Address limitations explicitly (e.g., biases in public databases toward certain taxa, challenges with repeat-rich assemblies, potential homoplasy in short motifs).
