# Supplementary material for: Evolutionary patterns of the mitochondrial control region in vertebrates: A large-scale comparative analysis
Source: PLoS One. 2026 Jul 10;21(7):e0353555. doi: 10.1371/journal.pone.0353555 (PMC13353940; doi:10.1371/journal.pone.0353555)
Supplement: Attachment — Submitted filename: Response to reviwevers.docx [file pone.0353555.s012.docx]

Comments to the Editor:

The manuscript (Manuscript PONE-D-25-58380): "The evolution of the control region in the mitochondrial genome of vertebrates" addresses an important topic—the evolutionary dynamics of the mitochondrial control region (CR) across vertebrates—using a large dataset of 5,235 complete CR sequences spanning 12 classes. This scale exceeds many prior taxon-specific or smaller-scale analyses, and the focus on repetitive elements, conserved motifs (ETAS/TAS, CSBs), nucleotide composition, length variation, and duplications is relevant to mitogenome evolution, replication/transcription regulation, and lineage-specific diversification. The assembly and analysis of thousands of vertebrate CRs is ambitious and provides a valuable broad phylogenetic framework. The identification of lineage-specific patterns (e.g., longer/more variable CRs in tetrapods, especially amphibians and reptiles, vs. fishes; CR duplications largely restricted to certain reptiles and birds; correlations between CR length and tandem repeat abundance) offers useful empirical insights. The discussion of mechanisms like slipped-strand mispairing, replication slippage, and life-history influences on repeat accumulation is conceptually sound.

However, the manuscript has substantial issues that undermine its current suitability for publication in PLOS ONE. These concern methods transparency, reproducibility, data handling and potential errors, analytical rigor, structure and writing, and presentation of results. Many sections read as an early draft with inconsistencies, incomplete descriptions. Overall, the core idea and dataset have strong potential for PLOS ONE, which values large-scale comparative studies with broad relevance. However, major revisions are essential to elevate methodological rigor, fix errors, enhance transparency/reproducibility, and polish the presentation. I recommend resubmission after these issues are comprehensively addressed; a revised version could make a solid contribution to the field.

**Response to the Editor:** We sincerely thank the Editor for the careful evaluation of our manuscript and for the constructive and insightful comments provided. We greatly appreciate the recognition of the scope and relevance of our study, as well as the detailed recommendations aimed at improving the quality, rigor, and clarity of the manuscript.

In response to the Editor’s concerns and the comments from the reviewers, we have thoroughly revised the manuscript. Specifically, we have improved the description of the methodology, enhanced the transparency and reproducibility of the analyses, corrected identified inconsistencies and errors, expanded the explanation of data handling procedures, strengthened the analytical framework, and substantially revised the presentation and discussion of the results. We have also carefully edited the text to improve its structure, clarity, and readability.

All comments have been addressed point-by-point in the response document, and the corresponding modifications have been highlighted in the revised manuscript. We believe that these revisions have significantly strengthened the study and have improved its overall quality.

We are grateful for the Editor’s and reviewers’ valuable suggestions, which have greatly contributed to improving our work.

Comments to authors:

Main comments

- I would congratulate to authors for performing such a broad study with thousands of CRs sequences. I believe the manuscript will benefit much from the provided comments and well fit for the publication after revision. The provided text lacks a dedicated, detailed Methods section. Key elements are missing or only referred to: exact search strategy and filters used in GenBank/NCBI to retrieve the 5,235 "complete" CR sequences; criteria for defining "complete" CRs (critical given known assembly difficulties with repeats); bioinformatics pipeline for extraction, annotation of TAS/ETAS, central domain (CSB-A to F), and Domain III (CSB1-3); tools and parameters for motif discovery, consensus sequence generation, maximum-likelihood motif analysis (S5 Fig.), repeat detection (e.g., tandem repeat finder settings), statistical tests (Kruskal-Wallis, Dunn's with Bonferroni), and correlation analyses.

**Response:** We thank the reviewer for this positive assessment of our work and for the constructive suggestions provided. We agree that the original manuscript did not contain sufficient methodological detail regarding data retrieval, sequence selection, motif identification, repeat analyses, and statistical procedures.

In response, we have substantially revised and expanded the Methods section, which has now been reorganized into clearly defined subsections describing: The first subsection related of CR sequences of vertebrate species which includes (i) the **Dataset retrieval** of mitochondrial control region sequences from GenBank/NCBI, including the search strategy and filtering criteria; (ii) the **inclusion and exclusion criteria** used to define complete control regions; (iii) **Identification of putative CRs in unannotated genomes and quality filtering of CR sequences**; and (iv) **Detection of duplication events**. A second methodological part of Molecular characterization of the CR which includes (i) **Global CR characterization**; (ii) **Detection of Tandem Repeats**; (iii) **Identification of Extended Termination-Associated sequences (TAS/ETAS) region**; (iv) **Annotation of conserved sequences blocks (CSBs)**; and (v) **Alignment, comparative and phylogenetic analysis of CSB motifs.** These additions considerably improve the transparency, reproducibility, and methodological rigor of the study. The revised Methods section can be found in lines 101–200 of the revised manuscript.

- How were partial or ambiguous CRs excluded? How were duplicates or heteroplasmy handled? Describe quality filtering, multiple sequence alignment (if used), and any custom scripts for domain identification. Without this, results are not reproducible.

**Response:** We thank the reviewer for highlighting the need for additional details regarding sequence filtering and reproducibility. In the revised manuscript, we have expanded the Materials and Methods section to clarify the procedures used for sequence selection, quality control, alignment, and domain identification (see lines 111 to 141).

Specifically, we now describe the criteria used to exclude incomplete or ambiguous control regions, including the retention of only complete mitochondrial genomes, the exclusion of records with uncertain control-region annotations, and the removal of putative control regions shorter than 300 bp. We have also clarified the procedure used to identify control regions in previously unannotated genomes through comparative genomic analyses and multiple sequence alignments (see lines 130 to 141 and 797 to 806). We have further clarified that heteroplasmy was not explicitly assessed because analyses were performed on assembled mitochondrial genome sequences available in GenBank, which represent consensus assemblies rather than raw sequencing data (see lines 118 to 120).

In addition, we have expanded the description of multiple sequence alignment procedures, motif detection workflows, and the identification of TAS/ETAS and CSB domains. The role of the custom Python scripts used for motif coordinate extraction has also been clarified (See lines 148 to 200)

- The manuscript's primary strength is its ambitious scale—analyzing over 5,000 vertebrate CR sequences—which enables robust documentation of inter- and intra-class variation in length, repeat content, nucleotide composition, and conserved motif copy number/position. This provides a valuable empirical foundation for understanding how tandem repeat proliferation drives CR expansion in tetrapods while core regulatory elements (TAS, CSBs) show differential conservation, revealing lineage-specific evolutionary trajectories (e.g., elongation in anurans/reptiles, streamlining in birds/mammals). When methods are fully detailed, this work could serve as a useful reference for mitogenomic researchers. Therefore, major weaknesses include insufficient methodological transparency (no detailed pipeline for sequence retrieval, CR annotation, or motif detection), numerous numerical inconsistencies and typographical errors suggesting the manuscript is not yet polished, and limited reproducibility due to incomplete data sharing. Analytical descriptions for custom motifs and phylogenetic motif analyses need expansion, the novelty claim requires better contextualization against existing literature, and the text contains structural/grammatical issues. These issues currently compromise rigor and clarity.

**Response:** We thank the Editor for the careful evaluation of our manuscript and for recognizing the value of the large-scale comparative dataset assembled in this study. We appreciate the constructive comments regarding methodological transparency, reproducibility, data presentation, and manuscript clarity.

In response, we have substantially revised the manuscript. The Materials and Methods section has been extensively expanded and reorganized to provide a detailed and reproducible description of the analytical workflow, including sequence retrieval and filtering procedures, control region identification and annotation, tandem repeat detection, TAS/ETAS identification, conserved sequence block (CSB) annotation, and statistical analyses. We now provide explicit descriptions of the software, parameters, motif definitions, similarity thresholds, and hierarchical search strategies used throughout the study. In addition, we clarify the distinction between reference-based and homology-guided annotation approaches and provide supplementary datasets containing motif coordinates and annotation results (See lines 148 to 188 of the revised manuscript).

We have also carefully reviewed the manuscript to correct numerical inconsistencies, typographical errors, and grammatical issues, and we have improved the overall structure and clarity of the text. Furthermore, we expanded the description of the motif-based comparative analyses and better contextualized the novelty and significance of our findings in relation to previous large-scale and taxon-specific studies of vertebrate mitochondrial control regions. Finally, we have clarified the purpose and scope of the phylogenetic analyses, which were used to evaluate clustering patterns of CSB variants rather than to infer deep evolutionary relationships (see lines 189 to 200 of the revised manuscript). We believe that these revisions have substantially improved the rigor, transparency, reproducibility, and presentation of the manuscript, and we thank the Editor and reviewers for their valuable suggestions.

Require thorough proofreading and verification:

- Abstract: "5,235 complete vertebrate CRs spanning 12 classes"; "tetrapods (1,283 ± 239.5 bp) than in fishes (969 ± 489.6 bp)". Later text reverses or varies SDs (e.g., fishes 969.25 ± 239.5; tetrapods 1,283.27 ± 489.6). Fish vs. tetrapod numbers shift slightly across pages.

**Response:** We thank the reviewer for identifying these inconsistencies. Upon re-examination of the manuscript, we found that the reported mean control region (CR) lengths for fishes and tetrapods were correct, but in some sections the associated standard deviation values were inadvertently transposed during manuscript preparation (See lines 19 to 20 of the revised manuscript).

- "11 vertebrate taxonomic classes" in one place, "12" in another; "8 taxonomic classes" for fishes.

**Response**: We thank the reviewer for identifying this inconsistency. After reviewing the manuscript, we confirmed that the dataset comprises 5,235 complete vertebrate control regions representing 11 taxonomic classes (See line 14). The references to "12 classes" and other inconsistent class counts were typographical errors introduced during manuscript preparation.

- Minimum length 329 bp in Percopsis transmontana (note spelling: transmontana).

**Response:** We thank the Editor for identifying this typographical error. The species name has been corrected from Percopsis transmotana to Percopsis transmontana in the revised manuscript (lines 205 and 217).

- Duplication frequencies: Reptiles 35.3% (108/306), Aves 20.16% (158/767), Amphibia 7.1% (17/241)—verify totals against overall 2,374 tetrapod sequences.

**Response:** We thank the Editor for requesting verification of the duplication frequencies and sample sizes. We have carefully rechecked all calculations and confirmed that the reported values are correct. A total of 283 CR duplication events were identified among the 2,374 tetrapod sequences analyzed, corresponding to an overall duplication frequency of 11.9%. These duplications were distributed among reptiles (108/306; 35.3%), birds (158/767; 20.6%), and amphibians (17/241; 7.1%), while no duplication events were detected in mammals. We have revised the text to clarify the relationship between the class-specific frequencies and the overall tetrapod dataset and verified that all percentages and sample sizes are reported consistently throughout the manuscript (See lines 169 to 171 of the revised manuscript).

- Check the correct spelling of the scientific name and pay attention to the inconsistent capitalization and italics for taxa.

**Response:** We thank the Editor for highlighting these nomenclatural and formatting inconsistencies. We have carefully reviewed the entire manuscript and corrected misspelled scientific names, including Percopsis transmontana, where applicable. In addition, we standardized the formatting of taxonomic names throughout the manuscript, ensuring consistent use of italics for genus and species names and uniform capitalization of taxonomic ranks in accordance with journal style guidelines. These corrections have been implemented throughout the main text, figures, tables, figure legends, and supplementary materials.

Analytical rigor and interpretation:

- Phylogenetic context for motif evolution (S5 Fig., ML analysis of consensus motifs) is promising but requires clearer methods (alignment, model selection, tree inference) and discussion of limitations (e.g., short motifs, saturation).

**Response**: We thank the Editor for this valuable suggestion. To improve methodological transparency, we have expanded the description of the phylogenetic analyses performed on CSB consensus motifs. The revised Methods section now provides additional details regarding sequence alignment, tree reconstruction procedures, bootstrap support, and the criteria used to evaluate motif relationships. We have also clarified that these analyses were intended to investigate broad patterns of motif similarity among vertebrate lineages rather than to infer species-level evolutionary relationships (See lines 189 to 200 of the revised manuscript).

In addition, we have expanded the Discussion to acknowledge the limitations of phylogenetic analyses based on short conserved motifs, including the limited number of informative sites, potential homoplasy, and the possibility of substitution saturation. We now explicitly caution against interpreting these motif-based trees as organismal phylogenies and instead present them as exploratory analyses of motif evolution and conservation (See lines 718 to 730 of the revised manuscript).

- Nucleotide skews and compositional biases: Discuss potential artifacts from replication asymmetry or sequencing/assembly biases in repeat-rich regions.

**Response**: We thank the Editor for this important suggestion. We have expanded the Discussion to consider both biological and technical factors that may influence nucleotide skews and compositional biases in vertebrate control regions. Specifically, we now discuss the potential role of replication asymmetry in generating strand-specific mutational biases, as well as the possibility that repeat-rich regions may be more susceptible to sequencing and assembly artifacts. We also clarify that sequences with ambiguous control-region annotations were excluded from the analyses and that the large-scale consistency of the observed patterns across vertebrate lineages supports their biological relevance conservation (See lines 568 to 583 of the revised manuscript).

- Duplications: Clarify detection method and functional implications (e.g., concerted evolution in snakes/birds).

**Response:** We thank the reviewer for this valuable suggestion. We have revised the manuscript to clarify both the detection of CR duplications and their potential functional significance (See lines 142 to 145 of the revised manuscript).

- Strengthen the "why these matters" section: Link findings more explicitly to broader questions in mitochondrial evolution, compensatory evolution, or implications for phylogenomics/barcoding (CR is often excluded due to variability).

**Response:** We thank the reviewer for this suggestion. These broader implications were addressed throughout the revised Discussion. Specifically, each subsection links the observed patterns of CR variation to broader questions in mitochondrial genome evolution, including the roles of regulatory motif turnover, tandem repeat expansion, CR duplication, and compensatory evolution in shaping mitochondrial genome architecture. We also expanded the Discussion and Conclusion (highlighted in the text) to provide a more concise synthesis of these themes, emphasizing how lineage-specific remodeling of the control region reflects the interplay between functional constraint and evolutionary innovation. Additionally, we highlight the relevance of our findings for comparative mitochondrial genomics and phylogenetic studies, given the extensive structural variability of the CR across vertebrates and the identification of conserved regulatory elements that persist despite substantial sequence divergence.

Writing and presentation:

- Title is clear but could specify "large-scale comparative analysis" or similar for precision. Claims of "first comprehensive comparative framework" need tempering—while the scale is large, prior comparative mitogenomic work exists (including on CR in specific clades or broader vertebrate mitogenomes). Position the study more accurately as a large-scale synthesis rather than wholly unprecedented.

**Response:** We thank the reviewer for this suggestion. We agree that the original title could overstate the novelty of the study. To better reflect the scope and comparative nature of our work, we have revised the title to emphasize the large-scale comparative framework and synthesis aspect of the analysis, while avoiding claims of being wholly unprecedented (see lines 1 and 2).

- Please write all the terms in full form when they appear at the first time; e. g ETAS: Extended Termination-Associated Sequences in Abstract

**Response:** We thank the reviewer for this observation. We have carefully revised the manuscript to ensure that all abbreviations and acronyms are defined in full at their first occurrence, including in the Abstract (see lines 14 to 15).

- The provided text is too lengthy, fragmented with repetitive phrasing or long sentences. Please improve its fluency, use concise scientific English and avoid redundancy.

Response: We thank the reviewer for this valuable suggestion. We have thoroughly revised the manuscript to improve readability, fluency, and overall conciseness. Redundant statements were removed, repetitive phrasing was minimized, and several long or fragmented sentences were rewritten using clearer scientific language. The Results and Discussion sections were streamlined to improve logical flow while retaining all relevant information and interpretations. As a result, the manuscript was reduced from 880 to 764 lines (excluding references), despite the expansion of the Materials and Methods section and several discussion sections in response to the editor's and reviewers' comments. These revisions have substantially improved the clarity, conciseness, and readability of the manuscript.

• Provide a complete, standalone ‘Methods section’ with all bioinformatics details, parameters, and validation steps. Deposit data/code publicly and update the Data Availability statement with specific links/DOIs.

**Response:** We thank the reviewer for this important suggestion. We have substantially revised the Materials and Methods section to provide a complete and self-contained description of all analytical procedures. The revised Methods now include detailed information on sequence retrieval, data filtering, motif identification, repeat detection, duplication analyses, statistical procedures, software packages, and parameter settings used throughout the study. We also added validation and quality-control procedures where applicable to improve transparency and reproducibility. In addition, we revised the Data Availability Statement to provide direct access to all datasets used in this study. The complete list of mitochondrial genomes analyzed, accession numbers, and supplementary datasets are now provided in the manuscript and supplementary materials (see lines 100 to 200). These changes ensure that the study is fully reproducible and that all data and analytical resources are publicly accessible.

• Systematically verify and standardize all numerical results, and statistical reporting across abstract, results, tables, and figures. Include sample sizes (n) for all means ± SD and post-hoc tests. Several times ‘±’ is missing when average (mean)±SD are reported. E.g. Page 9, Line 57: ‘between 1,028 900 to 1,581 bp in Aves’/ Page 22, Line 345: and 2.01 1.0477 copies per…

**Response:** We thank the reviewer for identifying these inconsistencies. We have carefully rechecked all numerical values, statistical results, and summary statistics throughout the manuscript, including the Abstract, Results, Tables, Figures, and Supplementary Materials. Means, standard deviations, sample sizes (n), test statistics, p-values, and post hoc comparisons were systematically verified for consistency across all sections. In addition, we standardized the reporting format of descriptive statistics throughout the manuscript, ensuring that all mean values are accompanied by their corresponding standard deviations (mean ± SD) and sample sizes where appropriate. We also corrected formatting inconsistencies, including instances where the symbol “±” was inadvertently omitted (e.g., Page 3, Line 50; Page 18, Line 399 of the revised manuscript), and revised all statistical reporting to follow a uniform style. These changes improve accuracy, clarity, and consistency throughout the manuscript.

• Please be consistent with format when reporting standard deviation: use +/- OR ± throughout the text; e.g. Page 12, Line 122: CR length was 969.25 (+/- 239.5) bp/ replace Fig 1a ----> Fig 1A

**Response**: We thank the reviewer for pointing out these formatting inconsistencies. We have carefully reviewed the entire manuscript and standardized the reporting of standard deviations using the symbol “±” throughout the text, tables, figure legends, and supplementary materials. All instances of alternative formats (e.g., “+/-”) were corrected to ensure consistency. For example, the expression “CR length was 969.25 (+/- 239.5) bp” has been revised to “CR length was 969.25 ± 239.5 bp” (see Page 10, Line 216 of the revised manuscript).

In addition, we standardized the formatting of figure citations throughout the manuscript, ensuring uniform use of uppercase panel labels (e.g., “Fig. 1A” instead of “Fig. 1a”) (see Page 10, Line 207 of the revised manuscript). Similar corrections were applied wherever necessary to maintain consistency and improve readability.

• Proofread thoroughly for grammar, typos, consistency (e.g., GC- vs. CG-, CSB- vs. CBS, fish vs. tetrapod stats), and flow. Consider professional editing if needed. E.g. Page 15, Line 193: statistically significant lower GC content...; replace 'significantly' with 'significant'/ Page 17, Line 239: delete ‘the’ a posteriori… and I would reword it as follow: “… and a posteriori Dunn's test indicated significant variation among all four compared classes..”

**Response**: We thank the reviewer for these careful observations. We have thoroughly proofread the entire manuscript to improve grammar, spelling, consistency, and overall readability. Particular attention was given to the consistent use of terminology and abbreviations (e.g., GC-content, GC-skew, CSB, coreTAS, fish versus tetrapod comparisons), as well as to the uniform reporting of statistical results throughout the text. (e.g. Page 14, Line 289 of the revised manuscript). We also implemented the specific corrections suggested by the reviewer. For example, expressions such as “statistically significant lower GC-content” were revised to “significantly lower GC-content” or “significant differences in GC-content,” as appropriate, and grammatical issues such as the unnecessary use of “the” before “a posteriori” were corrected.( e.g. Page 14, Line 292 of the revised manuscript)

• Page 18, Line 250: Testudines

**Response**: We thank the reviewer for identifying this typographical inconsistency. The term on Page 15, Line 326 has been corrected to Testudines.

• Page 19, Line 290: the CR length was moderately correlated …

**Response:** We thank the reviewer for this observation. The phrase on Page 19, Line 290 (preview version in discussion section) was removed during revision to improve clarity and avoid redundant wording. We also reviewed similar expressions throughout the manuscript to ensure concise and consistent reporting of statistical results.

• Page 23, Line 372: frequently detected (74%)…

**Response:** We thank the reviewer for this observation. The phrase on Page 19, Line 419 was modified to “CSB-D was detected in 74% of sequences”.

• Page 24, Line 381: Delete "in", also "ing": ...are lacking CSB-F to CSB-B within their CRs.

**Response:** We thank the reviewer for this correction. The sentence has been revised accordingly, and the unnecessary words (“in” and “ing”) have been removed to improve grammatical clarity. The corrected sentence now reads: “...are lacking CSB-F to CSB-B within their CRs.” (See Page 20, Line 428 of the revised manuscript)

• Page 32, Line 579: These partial copies retain an important? Please check the sentence for its structure and meaning.

**Response:** We thank the reviewer for pointing out this issue. The sentence has been revised to correct its structure and improve clarity. The phrase “important” was incomplete/unclear in the original version, and the sentence has been rewritten accordingly to accurately reflect the intended meaning. Revised sentence: “These partial copies retain a high sequence similarity (~70%) to the original region but span only 30–60% of its length, suggesting relatively recent duplication events followed by limited sequence divergence…” (See Page 27, Lines 591 to 593 of the revised manuscript)

• Ensure all figures/tables are complete, high-resolution, and self-explanatory with proper legends! In current version, provided figures are in low quality I cannot read the words.

**Response:** We thank the reviewer for this important comment. We have carefully reviewed all figures and tables and replaced them with high-resolution versions to ensure clarity and readability. All figures have been updated to improve visual quality, and the legends have been revised to make them fully self-explanatory. We confirm that all labels and text are now legible in the revised manuscript.

• Address limitations explicitly (e.g., biases in public databases toward certain taxa, challenges with repeat-rich assemblies, potential homoplasy in short motifs).

**Response:** We thank the reviewer for this valuable suggestion. The limitations raised, including potential biases in public databases toward certain taxa, challenges associated with repeat-rich region assemblies, and the possibility of homoplasy in short motifs, have been explicitly addressed and incorporated into the revised Discussion section of the manuscript.
