# Supplementary material for: Evolutionary patterns of the mitochondrial control region in vertebrates: A large-scale comparative analysis
Source: PLoS One. 2026 Jul 10;21(7):e0353555. doi: 10.1371/journal.pone.0353555 (PMC13353940; doi:10.1371/journal.pone.0353555)
Supplement: Peer review history

# Peer review: Decision Letter 0

**DOI:** 10.1371/journal.pone.0353555.r001

**Authors:** Amaal Gh. Yasser

17 May 2026

PONE-D-25-58380

The evolution of the control region in the mitochondrial genome of vertebrates

PLOS One

Dear Dr.

Prada Quiroga,

Thank you for submitting your manuscript to PLOS ONE. After careful consideration, we feel that it has merit but does not fully meet PLOS ONE’s publication criteria as it currently stands. Therefore, we invite you to submit a revised version of the manuscript that addresses the points raised during the review process.

==============================

After careful consideration and evaluation of the reviewers’ comments, we believe that the manuscript addresses an interesting and important topic. However, the current version of the manuscript cannot be accepted for publication at this stage. We invite you to revise the manuscript substantially and resubmit it for further consideration.

Please carefully address all reviewers’ comments and provide a detailed response explaining how each point has been addressed in the revised version.

==============================

Please submit your revised manuscript by Jul 01 2026 11:59PM. If you will need more time than this to complete your revisions, please reply to this message or contact the journal office at plosone@plos.org. When you're ready to submit your revision, log on to https://www.editorialmanager.com/pone/ and select the 'Submissions Needing Revision' folder to locate your manuscript file.

Please include the following items when submitting your revised manuscript:

- A letter that responds to each point raised by the academic editor and reviewer(s). You should upload this letter as a separate file labeled 'Response to Reviewers'.
- A marked-up copy of your manuscript that highlights changes made to the original version. You should upload this as a separate file labeled 'Revised Manuscript with Track Changes'.
- An unmarked version of your revised paper without tracked changes. You should upload this as a separate file labeled 'Manuscript'.

If you would like to make changes to your financial disclosure, please include your updated statement in your cover letter. Guidelines for resubmitting your figure files are available below the reviewer comments at the end of this letter.

If applicable, we recommend that you deposit your laboratory protocols in protocols.io to enhance the reproducibility of your results. Protocols.io assigns your protocol its own identifier (DOI) so that it can be cited independently in the future. For instructions see: https://journals.plos.org/plosone/s/submission-guidelines#loc-laboratory-protocols. Additionally, PLOS ONE offers an option for publishing peer-reviewed Lab Protocol articles, which describe protocols hosted on protocols.io. Read more information on sharing protocols at https://plos.org/protocols?utm_medium=editorial-email&utm_source=authorletters&utm_campaign=protocols.

As the corresponding author, your ORCID iD is verified in the submission system and will appear in the published article. PLOS supports the use of ORCID, and we encourage all coauthors to register for an ORCID iD and use it as well. Please encourage your coauthors to verify their ORCID iD within the submission system before final acceptance, as unverified ORCID iDs will not appear in the published article. *Only*  the individual author can complete the verification step; PLOS staff *cannot*  verify ORCID iDs on behalf of authors.

We look forward to receiving your revised manuscript.

Kind regards,

Amaal Gh. Yasser, Ph.D.

Academic Editor

PLOS One

Journal Requirements:

When submitting your revision, we need you to address these additional requirements.

1. Please ensure that your manuscript meets PLOS ONE's style requirements, including those for file naming. The PLOS ONE style templates can be found at

https://journals.plos.org/plosone/s/file?id=wjVg/PLOSOne_formatting_sample_main_body.pdf and

https://journals.plos.org/plosone/s/file?id=ba62/PLOSOne_formatting_sample_title_authors_affiliations.pdf

2. Please note that PLOS One has specific guidelines on code sharing for submissions in which author-generated code underpins the findings in the manuscript. In these cases, we expect all author-generated code to be made available without restrictions upon publication of the work. Please review our guidelines at https://journals.plos.org/plosone/s/materials-and-software-sharing#loc-sharing-code and ensure that your code is shared in a way that follows best practice and facilitates reproducibility and reuse.

3. We note that the grant information you provided in the ‘Funding Information’ and ‘Financial Disclosure’ sections do not match.

When you resubmit, please ensure that you provide the correct grant numbers for the awards you received for your study in the ‘Funding Information’ section.

4. Thank you for stating the following financial disclosure:

“This study is supported by the Oficina de Investigaciones of the University of Tolima.”

Please state what role the funders took in the study. If the funders had no role, please state: "The funders had no role in study design, data collection and analysis, decision to publish, or preparation of the manuscript."

If this statement is not correct you must amend it as needed.

Please include this amended Role of Funder statement in your cover letter; we will change the online submission form on your behalf.

5. If the reviewer comments include a recommendation to cite specific previously published works, please review and evaluate these publications to determine whether they are relevant and should be cited. There is no requirement to cite these works unless the editor has indicated otherwise.

**Additional Editor Comments:**

Dear Author,

Thank you for submitting your manuscript to PLOS ONE.

After careful consideration and evaluation of the reviewers’ comments, we believe that the manuscript addresses an interesting and important topic. However, the current version of the manuscript cannot be accepted for publication at this stage. We invite you to revise the manuscript substantially and resubmit it for further consideration.

Please carefully address all reviewers’ comments and provide a detailed response explaining how each point has been addressed in the revised version.

We appreciate your contribution to the journal and look forward to receiving your revised manuscript.

Kind regards,

Amaal.

Reviewers' comments:

Reviewer's Responses to Questions

**Comments to the Author**

1. Is the manuscript technically sound, and do the data support the conclusions?

The manuscript must describe a technically sound piece of scientific research with data that supports the conclusions. Experiments must have been conducted rigorously, with appropriate controls, replication, and sample sizes. The conclusions must be drawn appropriately based on the data presented.

Reviewer #1: Yes

Reviewer #2: Yes

**********

2. Has the statistical analysis been performed appropriately and rigorously?

Reviewer #1: Yes

Reviewer #2: Yes

**********

3. Have the authors made all data underlying the findings in their manuscript fully available?

The PLOS Data policy requires authors to make all data underlying the findings described in their manuscript fully available without restriction, with rare exception (please refer to the Data Availability Statement in the manuscript PDF file). The data should be provided as part of the manuscript or its supporting information, or deposited to a public repository. For example, in addition to summary statistics, the data points behind means, medians and variance measures should be available. If there are restrictions on publicly sharing data—e.g. participant privacy or use of data from a third party—those must be specified.

Reviewer #1: Yes

Reviewer #2: Yes

**********

4. Is the manuscript presented in an intelligible fashion and written in standard English?

PLOS ONE does not copyedit accepted manuscripts, so the language in submitted articles must be clear, correct, and unambiguous. Any typographical or grammatical errors should be corrected at revision, so please note any specific errors here.

Reviewer #1: Yes

Reviewer #2: Yes

**********

5. Review Comments to the Author

Please use the space provided to explain your answers to the questions above. You may also include additional comments for the author, including concerns about dual publication, research ethics, or publication ethics. (Please upload your review as an attachment if it exceeds 20,000 characters)

Reviewer #1: This manuscript presents a large-scale comparative analysis of the mitochondrial control region (CR) across vertebrates using 5,235 complete sequences retrieved from GenBank. The authors investigate variation in CR length, repetitive elements, conserved sequence blocks (ETAS and CSBs), nucleotide composition, and duplication patterns across major vertebrate groups. The study aims to provide a comprehensive framework for understanding the evolution and structural variability of the mitochondrial control region.

The topic is relevant and timely, and the dataset analyzed is impressively large. The comparative perspective across multiple vertebrate classes provides useful insights into lineage-specific patterns in mitochondrial genome architecture. The manuscript contains extensive statistical analyses and attempts to link structural variation with evolutionary mechanisms.

However, despite the potential significance of the study, the manuscript currently suffers from several methodological and presentation issues that need clarification before it can be considered for publication. In particular, the bioinformatic workflow, sequence selection criteria, repeat detection methods, and motif identification procedures are insufficiently described, making it difficult to reproduce the analyses. Some interpretations are also overstated relative to the evidence presented, and the manuscript would benefit from clearer structure and improved figure interpretation.

Overall, the manuscript has scientific merit, but major revisions are required to improve methodological transparency, strengthen the interpretation of results, and enhance the clarity of the presentation.

The Methods section lacks sufficient detail to ensure reproducibility. Important information that should be clarified includes:1) How the 5,235 mitochondrial control regions were selected from GenBank. 2) Criteria used to determine “complete CR sequences”. 3) Whether sequences were manually curated or automatically extracted. The manuscript reports detection of repetitive elements and conserved motifs (ETAS and CSBs), but the algorithms or software used for identifying these motifs are not clearly described. The dataset contains 2,861 fish sequences and 2,374 tetrapod sequences, but the manuscript does not explain whether this distribution reflects: true biological diversity, availability of sequences in GenBank and deliberate sampling strategy. Because the dataset relies on public database sequences, the authors should discuss potential biases caused by uneven taxonomic representation or incomplete assemblies. The authors report a strong correlation between CR length and repeat abundance (r = 0.69). However, the manuscript sometimes implies causation, suggesting that repeat expansion drives CR evolution. The authors should be more cautious and clarify that: 1) correlation does not demonstrate causation. 2) other processes (e.g., recombination, replication dynamics) may contribute to CR length variation. Additional analyses (e.g., phylogenetically controlled tests) would strengthen the argument. Although the study includes many vertebrate taxa, the analyses are largely descriptive rather than phylogenetically explicit. For example: correlations and statistical comparisons are performed across classes, but phylogenetic non-independence is not considered. Because mitochondrial genome features may reflect shared ancestry, the authors should consider:1) phylogenetic comparative methods. 2) controlling for phylogenetic signal. Without this, some observed patterns may reflect phylogenetic clustering rather than evolutionary processes. The discussion frequently proposes adaptive explanations for CR variation (e.g., metabolic demands, thermoregulation, life-history traits). However, these interpretations are largely speculative and not supported by direct tests in this study. For instance: the hypothesis linking CR repeat expansion to metabolic plasticity in amphibians or flight energetics in birds should be clearly framed as hypotheses rather than demonstrated conclusions. The manuscript references numerous supplementary tables and figures (S1–S5), but their descriptions in the text are limited. The authors should: 1) ensure that all supplementary materials are clearly explained. 2) improve figure captions to describe statistical tests and sample sizes. 3) clarify which datasets correspond to each analysis. The Discussion is very long and sometimes repetitive. Several sections restate results already presented earlier. Condensing this section would improve readability.

The manuscript presents an interesting and potentially valuable comparative analysis of vertebrate mitochondrial control regions. However, substantial revisions are required to improve methodological transparency, clarify statistical interpretations, and moderate speculative explanations.

Addressing these issues would significantly strengthen the manuscript and improve its contribution to the field of mitochondrial genome evolution.

Reviewer #2: Comments to authors:

Main comments

- I would congratulate to authors for performing such a broad study with thousands of CRs sequences. I believe the manuscript will benefit much from the provided comments and well fit for the publication after revision. The provided text lacks a dedicated, detailed Methods section. Key elements are missing or only referred to: exact search strategy and filters used in GenBank/NCBI to retrieve the 5,235 "complete" CR sequences; criteria for defining "complete" CRs (critical given known assembly difficulties with repeats); bioinformatics pipeline for extraction, annotation of TAS/ETAS, central domain (CSB-A to F), and Domain III (CSB1-3); tools and parameters for motif discovery, consensus sequence generation, maximum-likelihood motif analysis (S5 Fig.), repeat detection (e.g., tandem repeat finder settings), statistical tests (Kruskal-Wallis, Dunn's with Bonferroni), and correlation analyses.

- How were partial or ambiguous CRs excluded? How were duplicates or heteroplasmy handled? Describe quality filtering, multiple sequence alignment (if used), and any custom scripts for domain identification. Without this, results are not reproducible.

- The manuscript's primary strength is its ambitious scale—analyzing over 5,000 vertebrate CR sequences—which enables robust documentation of inter- and intra-class variation in length, repeat content, nucleotide composition, and conserved motif copy number/position. This provides a valuable empirical foundation for understanding how tandem repeat proliferation drives CR expansion in tetrapods while core regulatory elements (TAS, CSBs) show differential conservation, revealing lineage-specific evolutionary trajectories (e.g., elongation in anurans/reptiles, streamlining in birds/mammals). When methods are fully detailed, this work could serve as a useful reference for mitogenomic researchers. Therefore, major weaknesses include insufficient methodological transparency (no detailed pipeline for sequence retrieval, CR annotation, or motif detection), numerous numerical inconsistencies and typographical errors suggesting the manuscript is not yet polished, and limited reproducibility due to incomplete data sharing. Analytical descriptions for custom motifs and phylogenetic motif analyses need expansion, the novelty claim requires better contextualization against existing literature, and the text contains structural/grammatical issues. These issues currently compromise rigor and clarity.

Require thorough proofreading and verification:

- Abstract: "5,235 complete vertebrate CRs spanning 12 classes"; "tetrapods (1,283 ± 239.5 bp) than in fishes (969 ± 489.6 bp)". Later text reverses or varies SDs (e.g., fishes 969.25 ± 239.5; tetrapods 1,283.27 ± 489.6). Fish vs. tetrapod numbers shift slightly across pages.

- "11 vertebrate taxonomic classes" in one place, "12" in another; "8 taxonomic classes" for fishes.

- Minimum length 329 bp in Percopsis transmontana (note spelling: transmontana).

- Duplication frequencies: Reptiles 35.3% (108/306), Aves 20.16% (158/767), Amphibia 7.1% (17/241)—verify totals against overall 2,374 tetrapod sequences.

- Check the correct spelling of the scientific name and pay attention to the inconsistent capitalization and italics for taxa.

Analytical rigor and interpretation:

- Phylogenetic context for motif evolution (S5 Fig., ML analysis of consensus motifs) is promising but requires clearer methods (alignment, model selection, tree inference) and discussion of limitations (e.g., short motifs, saturation).

- Nucleotide skews and compositional biases: Discuss potential artifacts from replication asymmetry or sequencing/assembly biases in repeat-rich regions.

- Duplications: Clarify detection method and functional implications (e.g., concerted evolution in snakes/birds).

- Strengthen the "why these matters" section: Link findings more explicitly to broader questions in mitochondrial evolution, compensatory evolution, or implications for phylogenomics/barcoding (CR is often excluded due to variability).

Writing and presentation:

- Title is clear but could specify "large-scale comparative analysis" or similar for precision. Claims of "first comprehensive comparative framework" need tempering—while the scale is large, prior comparative mitogenomic work exists (including on CR in specific clades or broader vertebrate mitogenomes). Position the study more accurately as a large-scale synthesis rather than wholly unprecedented.

- Please write all the terms in full form when they appear at the first time; e. g ETAS: Extended Termination-Associated Sequences in Abstract

- The provided text is too lengthy, fragmented with repetitive phrasing or long sentences. Please improve its fluency, use concise scientific English and avoid redundancy.

• Provide a complete, standalone ‘Methods section’ with all bioinformatics details, parameters, and validation steps. Deposit data/code publicly and update the Data Availability statement with specific links/DOIs.

• Systematically verify and standardize all numerical results, and statistical reporting across abstract, results, tables, and figures. Include sample sizes (n) for all means ± SD and post-hoc tests. Several times ‘±’ is missing when average (mean)±SD are reported. E.g. Page 9, Line 57: ‘between 1,028 900 to 1,581 bp in Aves’/ Page 22, Line 345: and 2.01 1.0477 copies per…

• Please be consistent with format when reporting standard deviation: use +/- OR ± throughout the text; e.g. Page 12, Line 122: CR length was 969.25 (+/- 239.5) bp/ replace Fig 1a ----> Fig 1A

• Proofread thoroughly for grammar, typos, consistency (e.g., GC- vs. CG-, CSB- vs. CBS, fish vs. tetrapod stats), and flow. Consider professional editing if needed. E.g. Page 15, Line 193: statistically significant lower GC content...; replace 'significantly' with 'significant'/ Page 17, Line 239: delete ‘the’ a posteriori… and I would reword it as follow: “… and a posteriori Dunn's test indicated significant variation among all four compared classes..”

• Page 18, Line 250: Testudines

• Page 19, Line 290: the CR length was moderately correlated …

• Page 23, Line 372: frequently detected (74%)…

• Page 24, Line 381: Delete "in", also "ing": ...are lacking CSB-F to CSB-B within their CRs.

• Page 32, Line 579: These partial copies retain an important? Please check the sentence for its structure and meaning.

• Ensure all figures/tables are complete, high-resolution, and self-explanatory with proper legends! In current version, provided figures are in low quality I cannot read the words.

• Address limitations explicitly (e.g., biases in public databases toward certain taxa, challenges with repeat-rich assemblies, potential homoplasy in short motifs).

**********

6. PLOS authors have the option to publish the peer review history of their article (what does this mean?). If published, this will include your full peer review and any attached files.

If you choose “no”, your identity will remain anonymous but your review may still be made public.

**Do you want your identity to be public for this peer review?**  For information about this choice, including consent withdrawal, please see our Privacy Policy.

Reviewer #1: No

Reviewer #2: No

**********

[NOTE: If reviewer comments were submitted as an attachment file, they will be attached to this email and accessible via the submission site. Please log into your account, locate the manuscript record, and check for the action link "View Attachments". If this link does not appear, there are no attachment files.]

To ensure your figures meet our technical requirements, please review our figure guidelines: https://journals.plos.org/plosone/s/figures

You may also use PLOS’s free figure tool, NAAS, to help you prepare publication quality figures: https://journals.plos.org/plosone/s/figures#loc-tools-for-figure-preparation.

NAAS will assess whether your figures meet our technical requirements by comparing each figure against our figure specifications.

Attachment Submitted filename: Comments to PONE-D-25-58380.pdf

# Author comment: Author response to Decision Letter 1

**DOI:** 10.1371/journal.pone.0353555.r002

11 Jun 2026

Comments to the Editor:

The manuscript (Manuscript PONE-D-25-58380): "The evolution of the control region in the mitochondrial genome of vertebrates" addresses an important topic—the evolutionary dynamics of the mitochondrial control region (CR) across vertebrates—using a large dataset of 5,235 complete CR sequences spanning 12 classes. This scale exceeds many prior taxon-specific or smaller-scale analyses, and the focus on repetitive elements, conserved motifs (ETAS/TAS, CSBs), nucleotide composition, length variation, and duplications is relevant to mitogenome evolution, replication/transcription regulation, and lineage-specific diversification. The assembly and analysis of thousands of vertebrate CRs is ambitious and provides a valuable broad phylogenetic framework. The identification of lineage-specific patterns (e.g., longer/more variable CRs in tetrapods, especially amphibians and reptiles, vs. fishes; CR duplications largely restricted to certain reptiles and birds; correlations between CR length and tandem repeat abundance) offers useful empirical insights. The discussion of mechanisms like slipped-strand mispairing, replication slippage, and life-history influences on repeat accumulation is conceptually sound.

However, the manuscript has substantial issues that undermine its current suitability for publication in PLOS ONE. These concern methods transparency, reproducibility, data handling and potential errors, analytical rigor, structure and writing, and presentation of results. Many sections read as an early draft with inconsistencies, incomplete descriptions. Overall, the core idea and dataset have strong potential for PLOS ONE, which values large-scale comparative studies with broad relevance. However, major revisions are essential to elevate methodological rigor, fix errors, enhance transparency/reproducibility, and polish the presentation. I recommend resubmission after these issues are comprehensively addressed; a revised version could make a solid contribution to the field.

Response to the Editor: We sincerely thank the Editor for the careful evaluation of our manuscript and for the constructive and insightful comments provided. We greatly appreciate the recognition of the scope and relevance of our study, as well as the detailed recommendations aimed at improving the quality, rigor, and clarity of the manuscript.

In response to the Editor’s concerns and the comments from the reviewers, we have thoroughly revised the manuscript. Specifically, we have improved the description of the methodology, enhanced the transparency and reproducibility of the analyses, corrected identified inconsistencies and errors, expanded the explanation of data handling procedures, strengthened the analytical framework, and substantially revised the presentation and discussion of the results. We have also carefully edited the text to improve its structure, clarity, and readability.

All comments have been addressed point-by-point in the response document, and the corresponding modifications have been highlighted in the revised manuscript. We believe that these revisions have significantly strengthened the study and have improved its overall quality.

We are grateful for the Editor’s and reviewers’ valuable suggestions, which have greatly contributed to improving our work.

Comments to authors:

Main comments

- I would congratulate to authors for performing such a broad study with thousands of CRs sequences. I believe the manuscript will benefit much from the provided comments and well fit for the publication after revision. The provided text lacks a dedicated, detailed Methods section. Key elements are missing or only referred to: exact search strategy and filters used in GenBank/NCBI to retrieve the 5,235 "complete" CR sequences; criteria for defining "complete" CRs (critical given known assembly difficulties with repeats); bioinformatics pipeline for extraction, annotation of TAS/ETAS, central domain (CSB-A to F), and Domain III (CSB1-3); tools and parameters for motif discovery, consensus sequence generation, maximum-likelihood motif analysis (S5 Fig.), repeat detection (e.g., tandem repeat finder settings), statistical tests (Kruskal-Wallis, Dunn's with Bonferroni), and correlation analyses.

Response: We thank the reviewer for this positive assessment of our work and for the constructive suggestions provided. We agree that the original manuscript did not contain sufficient methodological detail regarding data retrieval, sequence selection, motif identification, repeat analyses, and statistical procedures.

In response, we have substantially revised and expanded the Methods section, which has now been reorganized into clearly defined subsections describing: The first subsection related of CR sequences of vertebrate species which includes (i) the Dataset retrieval of mitochondrial control region sequences from GenBank/NCBI, including the search strategy and filtering criteria; (ii) the inclusion and exclusion criteria used to define complete control regions; (iii) Identification of putative CRs in unannotated genomes and quality filtering of CR sequences; and (iv) Detection of duplication events. A second methodological part of Molecular characterization of the CR which includes (i) Global CR characterization; (ii) Detection of Tandem Repeats; (iii) Identification of Extended Termination-Associated sequences (TAS/ETAS) region; (iv) Annotation of conserved sequences blocks (CSBs); and (v) Alignment, comparative and phylogenetic analysis of CSB motifs. These additions considerably improve the transparency, reproducibility, and methodological rigor of the study. The revised Methods section can be found in lines 101–200 of the revised manuscript.

- How were partial or ambiguous CRs excluded? How were duplicates or heteroplasmy handled? Describe quality filtering, multiple sequence alignment (if used), and any custom scripts for domain identification. Without this, results are not reproducible.

Response: We thank the reviewer for highlighting the need for additional details regarding sequence filtering and reproducibility. In the revised manuscript, we have expanded the Materials and Methods section to clarify the procedures used for sequence selection, quality control, alignment, and domain identification (see lines 111 to 141).

Specifically, we now describe the criteria used to exclude incomplete or ambiguous control regions, including the retention of only complete mitochondrial genomes, the exclusion of records with uncertain control-region annotations, and the removal of putative control regions shorter than 300 bp. We have also clarified the procedure used to identify control regions in previously unannotated genomes through comparative genomic analyses and multiple sequence alignments (see lines 130 to 141 and 797 to 806). We have further clarified that heteroplasmy was not explicitly assessed because analyses were performed on assembled mitochondrial genome sequences available in GenBank, which represent consensus assemblies rather than raw sequencing data (see lines 118 to 120).

In addition, we have expanded the description of multiple sequence alignment procedures, motif detection workflows, and the identification of TAS/ETAS and CSB domains. The role of the custom Python scripts used for motif coordinate extraction has also been clarified (See lines 148 to 200)

- The manuscript's primary strength is its ambitious scale—analyzing over 5,000 vertebrate CR sequences—which enables robust documentation of inter- and intra-class variation in length, repeat content, nucleotide composition, and conserved motif copy number/position. This provides a valuable empirical foundation for understanding how tandem repeat proliferation drives CR expansion in tetrapods while core regulatory elements (TAS, CSBs) show differential conservation, revealing lineage-specific evolutionary trajectories (e.g., elongation in anurans/reptiles, streamlining in birds/mammals). When methods are fully detailed, this work could serve as a useful reference for mitogenomic researchers. Therefore, major weaknesses include insufficient methodological transparency (no detailed pipeline for sequence retrieval, CR annotation, or motif detection), numerous numerical inconsistencies and typographical errors suggesting the manuscript is not yet polished, and limited reproducibility due to incomplete data sharing. Analytical descriptions for custom motifs and phylogenetic motif analyses need expansion, the novelty claim requires better contextualization against existing literature, and the text contains structural/grammatical issues. These issues currently compromise rigor and clarity.

Response: We thank the Editor for the careful evaluation of our manuscript and for recognizing the value of the large-scale comparative dataset assembled in this study. We appreciate the constructive comments regarding methodological transparency, reproducibility, data presentation, and manuscript clarity.

In response, we have substantially revised the manuscript. The Materials and Methods section has been extensively expanded and reorganized to provide a detailed and reproducible description of the analytical workflow, including sequence retrieval and filtering procedures, control region identification and annotation, tandem repeat detection, TAS/ETAS identification, conserved sequence block (CSB) annotation, and statistical analyses. We now provide explicit descriptions of the software, parameters, motif definitions, similarity thresholds, and hierarchical search strategies used throughout the study. In addition, we clarify the distinction between reference-based and homology-guided annotation approaches and provide supplementary datasets containing motif coordinates and annotation results (See lines 148 to 188 of the revised manuscript).

We have also carefully reviewed the manuscript to correct numerical inconsistencies, typographical errors, and grammatical issues, and we have improved the overall structure and clarity of the text. Furthermore, we expanded the description of the motif-based comparative analyses and better contextualized the novelty and significance of our findings in relation to previous large-scale and taxon-specific studies of vertebrate mitochondrial control regions. Finally, we have clarified the purpose and scope of the phylogenetic analyses, which were used to evaluate clustering patterns of CSB variants rather than to infer deep evolutionary relationships (see lines 189 to 200 of the revised manuscript). We believe that these revisions have substantially improved the rigor, transparency, reproducibility, and presentation of the manuscript, and we thank the Editor and reviewers for their valuable suggestions.

Require thorough proofreading and verification:

- Abstract: "5,235 complete vertebrate CRs spanning 12 classes"; "tetrapods (1,283 ± 239.5 bp) than in fishes (969 ± 489.6 bp)". Later text reverses or varies SDs (e.g., fishes 969.25 ± 239.5; tetrapods 1,283.27 ± 489.6). Fish vs. tetrapod numbers shift slightly across pages.

Response: We thank the reviewer for identifying these inconsistencies. Upon re-examination of the manuscript, we found that the reported mean control region (CR) lengths for fishes and tetrapods were correct, but in some sections the associated standard deviation values were inadvertently transposed during manuscript preparation (See lines 19 to 20 of the revised manuscript).

- "11 vertebrate taxonomic classes" in one place, "12" in another; "8 taxonomic classes" for fishes.

Response: We thank the reviewer for identifying this inconsistency. After reviewing the manuscript, we confirmed that the dataset comprises 5,235 complete vertebrate control regions representing 11 taxonomic classes (See line 14). The references to "12 classes" and other inconsistent class counts were typographical errors introduced during manuscript preparation.

- Minimum length 329 bp in Percopsis transmontana (note spelling: transmontana).

Response: We thank the Editor for identifying this typographical error. The species name has been corrected from Percopsis transmotana to Percopsis transmontana in the revised manuscript (lines 205 and 217).

- Duplication frequencies: Reptiles 35.3% (108/306), Aves 20.16% (158/767), Amphibia 7.1% (17/241)—verify totals against overall 2,374 tetrapod sequences.

Response: We thank the Editor for requesting verification of the duplication frequencies and sample sizes. We have carefully rechecked all calculations and confirmed that the reported values are correct. A total of 283 CR duplication events were identified among the 2,374 tetrapod sequences analyzed, corresponding to an overall duplication frequency of 11.9%. These duplications were distributed among reptiles (108/306; 35.3%), birds (158/767; 20.6%), and amphibians (17/241; 7.1%), while no duplication events were detected in mammals. We have revised the text to clarify the relationship between the class-specific frequencies and the overall tetrapod dataset and verified that all percentages and sample sizes are reported consistently throughout the manuscript (See lines 169 to 171 of the revised manuscript).

- Check the correct spelling of the scientific name and pay attention to the inconsistent capitalization and italics for taxa.

Response: We thank the Editor for highlighting these nomenclatural and formatting inconsistencies. We have carefully reviewed the entire manuscript and corrected misspelled scientific names, including Percopsis transmontana, where applicable. In addition, we standardized the formatting of taxonomic names throughout the manuscript, ensuring consistent use of italics for genus and species names and uniform capitalization of taxonomic ranks in accordance with journal style guidelines. These corrections have been implemented throughout the main text, figures, tables, figure legends, and supplementary materials.

Analytical rigor and interpretation:

- Phylogenetic context for motif evolution (S5 Fig., ML analysis of consensus motifs) is promising but requires clearer methods (alignment, model selection, tree inference) and discussion of limitations (e.g., short motifs, saturation).

Response: We thank the Editor for this valuable suggestion. To improve methodological transparency, we have expanded the description of the phylogenetic analyses performed on CSB consensus motifs. The revised Methods section now provides additional details regarding sequence alignment, tree reconstruction procedures, bootstrap support, and the criteria used to evaluate motif relationships. We have also clarified that these analyses were intended to investigate broad patterns of motif similarity among vertebrate lineages rather than to infer species-level evolutionary relationships (See lines 189 to 200 of the revised manuscript).

In addition, we have expanded the Discussion to acknowledge the limitations of phylogenetic analyses based on short conserved motifs, including the limited number of informative sites, potential homoplasy, and the possibility of substitution saturation. We now explicitly caution against interpreting these motif-based trees as organismal phylogenies and instead present them as exploratory analyses of motif evolution and conservation (See lines 718 to 730 of the revised manuscript).

- Nucleotide skews and compositional biases: Discuss potential artifacts from replication asymmetry or sequencing/assembly biases in repeat-rich regions.

Response: We thank the Editor for this important suggestion. We have expanded the Discussion to consider both biological and technical factors that may influence nucleotide skews and compositional biases in vertebrate control regions. Specifically, we now discuss the potential role of replication asymmetry in generating strand-specific mutational biases, as well as the possibility that repeat-rich regions may be more susceptible to sequencing and assembly artifacts. We also clarify that sequences with ambiguous control-region annotations were excluded from the analyses and that the large-scale consistency of the observed patterns across vertebrate lineages supports their biological relevance conservation (See lines 568 to 583 of the revised manuscript).

- Duplications: Clarify detection method and functional implications

Attachment Submitted filename: Response to reviwevers.docx

# Editor report: Decision Letter 1

**DOI:** 10.1371/journal.pone.0353555.r003

**Authors:** Amaal Gh. Yasser, Amaal Gh. Yasser

25 Jun 2026

Evolutionary patterns of the mitochondrial control region in vertebrates: a large-scale comparative analysis

PONE-D-25-58380R1

Dear Dr. Dr. Quiroga,

We’re pleased to inform you that your manuscript has been judged scientifically suitable for publication and will be formally accepted for publication once it meets all outstanding technical requirements.

Within one week, you’ll receive an e-mail detailing the required amendments. When these have been addressed, you’ll receive a formal acceptance letter and your manuscript will be scheduled for publication.

An invoice will be generated when your article is formally accepted. Please note, if your institution has a publishing partnership with PLOS and your article meets the relevant criteria, all or part of your publication costs will be covered. Please make sure your user information is up-to-date by logging into Editorial Manager at Editorial Manager® and clicking the ‘Update My Information' link at the top of the page. For questions related to billing, please contact billing support.

If your institution or institutions have a press office, please notify them about your upcoming paper to help maximize its impact. If they’ll be preparing press materials, please inform our press team as soon as possible -- no later than 48 hours after receiving the formal acceptance. Your manuscript will remain under strict press embargo until 2 pm Eastern Time on the date of publication. For more information, please contact onepress@plos.org.

Kind regards,

Amaal Gh. Yasser, Ph.D.

Academic Editor

PLOS One

Additional Editor Comments (optional):

Dear Dr. Quiroga,

Thank you for submitting the revised version of your manuscript and for the considerable effort invested in addressing the reviewers' comments and recommendations.

After careful evaluation of the revised manuscript and your detailed responses to the reviewers, I am pleased to inform you that your manuscript has been accepted for publication.

The revisions have substantially improved the quality and clarity of the manuscript, and the concerns raised during the peer-review process have been satisfactorily addressed.

On behalf of the editorial team, I would like to congratulate you on the acceptance of your work and thank you for choosing PLOS ONE for the dissemination of your research.

We appreciate your contribution and look forward to seeing your article published.

Congratulations once again, and we wish you continued success in your future research endeavors.

Kind regards,

Amaal Yasser

Academic Editor

Reviewers' comments:

# Editor report: Acceptance letter

**DOI:** 10.1371/journal.pone.0353555.r004

**Authors:** Amaal Gh. Yasser, Amaal Gh. Yasser

PONE-D-25-58380R1

PLOS One

Dear Dr. Prada-Quiroga,

I'm pleased to inform you that your manuscript has been deemed suitable for publication in PLOS One. Congratulations! Your manuscript is now being handed over to our production team.

At this stage, our production department will prepare your paper for publication. This includes ensuring the following:

* All references, tables, and figures are properly cited

* All relevant supporting information is included in the manuscript submission,

* There are no issues that prevent the paper from being properly typeset

You will receive further instructions from the production team, including instructions on how to review your proof when it is ready. Please keep in mind that we are working through a large volume of accepted articles, so please give us a few days to review your paper and let you know the next and final steps.

Lastly, if your institution or institutions have a press office, please let them know about your upcoming paper now to help maximize its impact. If they'll be preparing press materials, please inform our press team within the next 48 hours. Your manuscript will remain under strict press embargo until 2 pm Eastern Time on the date of publication. For more information, please contact onepress@plos.org.

You will receive an invoice from PLOS for your publication fee after your manuscript has reached the completed accept phase. If you receive an email requesting payment before acceptance or for any other service, this may be a phishing scheme. Learn how to identify phishing emails and protect your accounts at https://explore.plos.org/phishing.

If we can help with anything else, please email us at customercare@plos.org.

Thank you for submitting your work to PLOS ONE and supporting open access.

Kind regards,

PLOS ONE Editorial Office Staff

on behalf of

Dr. Amaal Gh. Yasser

Academic Editor

PLOS One
